# Enhancing postharvest food safety: the essential role of non-thermal technologies in combating fungal contamination and mycotoxins

**DOI:** 10.3389/fmicb.2025.1543716

**Published:** 2025-03-11

**Authors:** Junior Bernardo Molina-Hernandez, Carlos David Grande-Tovar, Lilia Neri, Johannes Delgado-Ospina, Massimiliano Rinaldi, Gustavo Adolfo Cordero-Bueso, Clemencia Chaves-López

**Affiliations:** ^1^Faculty of Bioscience and Technology for Food, Agriculture and Environment, University of Teramo, Teramo, Italy; ^2^Department of Agricultural and Food Sciences, University of Bologna, Cesena, Italy; ^3^Grupo de Investigación de Fotoquímica y Fotobiología, Universidad del Atlántico, Puerto Colombia, Colombia; ^4^Grupo de Investigación Biotecnología, Facultad de Ingeniería, Universidad de San Buenaventura Cali, Cali, Colombia; ^5^Department of Food and Drug, University of Parma, Parma, Italy; ^6^Laboratorio de Microbiología, CASEM, Dpto. Biomedicina, Biotecnología y Salud Pública, Universidad de Cádiz, Cádiz, Spain

**Keywords:** emerging technologies, post-harvest treatment, filamentous fungal suppression, reduction of mycotoxin, food safety, food preservation, food quality

## Abstract

During the production and storage of agricultural products, molds frequently occur as contaminants that can produce a wide range of secondary metabolites, the most important of which are mycotoxins. To solve these problems, the industry uses various methods, products and processes. This review examines the latest advances in novel non-thermal technologies for post-harvest inactivation of filamentous fungi and reduction of mycotoxins. These technologies include high pressure processes (HPP), ozone treatment, UV light, blue light, pulsed light, pulsed electric fields (PEF), cold atmospheric plasma (CAP), electron beams, ultrasound (US) and nanoparticles. Using data from previous studies, this review provides an overview of the primary mechanisms of action and recent results obtained using these technologies and emphasizes the limitations and challenges associated with each technology. The innovative non-thermal methods discussed here have been shown to be safe and efficient tools for reducing food mold contamination and infection. However, the effectiveness of these technologies is highly dependent on the fungal species and the structural characteristics of the mycotoxins. New findings related to the inactivation of fungi and mycotoxins underline that for a successful application it is essential to carefully determine and optimize certain key parameters in order to achieve satisfactory results. Finally, this review highlights and discusses future directions for non-thermal technologies. It emphasizes that they meet consumer demand for clean and safe food without compromising nutritional and sensory qualities.

## Graphical abstract

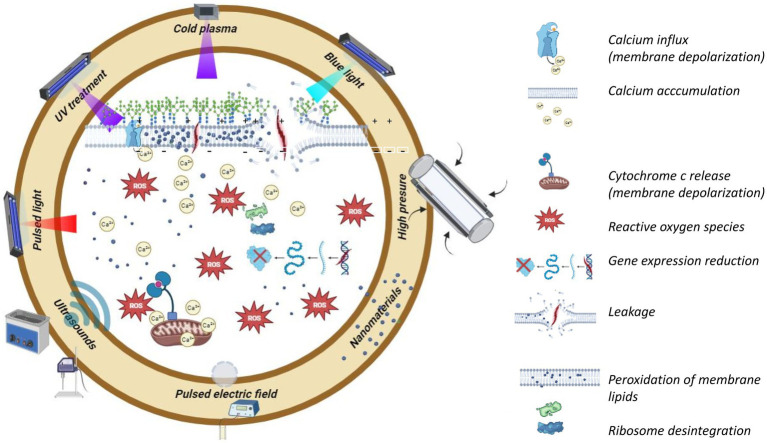

## 1 Introduction

The contamination of food and feed by fungi has become a global problem with a significant economic impact worldwide. Airborne fungal spores can easily infect, colonize, and spoil plants and food. The rapid spread and adaptability of the spores in different areas negates all active disease control strategies. In addition, the morphology and stress tolerance of fungal spores, as well as their production process and dormant state, are highly variable. It is estimated that fungi are responsible for losing up to 10–23% of crop products due to disease and additionally, 10 to 20% in the post-harvest processes (Stukenbrock and Gurr, 2023), and climate change can exacerbate the situation by creating conditions that make the occurrence and spread of foodborne hazards more likely because they weaken the resistance of host plants making them more susceptible to fungal diseases. On the other hand, it is possible that there will be a shift in mycotoxin-producing fungi and a change in global patterns of mycotoxin incidence (Casu et al., 2024; Delgado-Ospina et al., 2021). Mycotoxins are known to be dangerous secondary metabolites produced mostly by fungi of the genera *Aspergillus, Penicillium, Fusarium*, and *Alternaria.* Over 500 mycotoxins have been reported, with aflatoxins, ochratoxin A (OTA), fumonisins, trichothecenes, and zearalenone being the most significant due to their impact on agriculture, economics, and public health (Moretti et al., 2017). They form a chemically heterogeneous family, grouped together by their harmful effects on human and animal’s health, including permanent damage. Some of them are potentially mutagenic, teratogenic, carcinogenic, cytotoxic, neurotoxic, nephrotoxic, and estrogenic and contain immunosuppressive compounds (Laika et al., 2024a). European Food Safety Authority (EFSA) regularly publish reports on food safety issues, including mycotoxin-related recalls (www.efsa.europa.eu). According to Wu (2008), the average financial loss due to an EU border rejection ranges between €7,000 and €11,000. These expenses primarily cover administrative processes such as sampling, transportation, and storage, excluding additional costs related to the final disposal or management of contaminated goods. With climate change expected to increase natural toxin contamination, including mycotoxins, the resulting economic impact is anticipated to be even greater (Wu and Mitchell, 2016). Meanwhile, the European Union’s Rapid Alert System for Food and Feed (RASFF) reported that in 2023, mycotoxins ranked as the third most frequently notified hazard category. The majority of these cases involved the detection of aflatoxins, with nuts, nut products, and seeds being the most commonly affected product category. Mycotoxins can be transferred from a rotten part of vegetables and fruit with a high water content into the internal environment by diffusion. The concentration gradient of a penetrating substance through food is usually described by Fick’s second law and by the solutions of this differential equation (da Silva Lima et al., 2022). Furthermore, some studies have shown that the integrity of the fruit tissue and the vegetable tissue plays an essential role in the diffusion of mycotoxins; in addition, the skins of the fruit become more susceptible to increasing ripeness (Enikova et al., 2020). Mycotoxin exposure also occurs in animals regardless of the farming system—whether intensive, extensive, or mixed. Consequently, mycotoxin contamination poses a risk in nomadic and integrated crop-livestock systems that depend on forages, crops, and animal byproducts (El-Sayed et al., 2022). Although effective agricultural practices, quality control measures, and proper storage techniques help reduce the risk of mycotoxin exposure in industrialized nations, research indicates that several developing regions still face a significant threat, ranging from severe to extreme levels of contamination. For example, mycotoxins are highly prevalent in North and Central America, as well as South Asia, while other areas, including Southeast Asia, Oceania, and Europe, experience moderate to high levels of contamination (El-Sayed et al., 2022; Gruber-Dorninger et al., 2019).

Mycotoxins are highly resilient compounds that, once formed, are extremely difficult to eliminate from contaminated food and feed. They are chemically stable and can withstand various food processing methods, including high temperatures, freezing, and chemical treatments. Conventional decontamination techniques, such as washing, milling, or heating, often fail to completely remove them, and some methods may even lead to the formation of toxic byproducts (Awuchi et al., 2021). Consequently, controlling mycotoxins in food can be difficult and requires regular monitoring. Although preservatives and strict cleanliness in the factory (such as using a sterile line) can prevent spoilage by many fungal species, some fungi are highly resistant to preservatives and can persist even in storage conditions that typically inhibit unspecialized microbes. Therefore, alternatives other than antifungal agents must be available to control fungi and to avoid the emergence of resistant species. These alternatives must be able to prevent the proliferation of mycelia and the survival of spores and degrade mycotoxins that may be present in food. In this context, some alternatives to antimicrobials are thermal processing technologies, which are well established and relatively cost-effective, especially in large-scale production, and many types of food can be treated with these methods, from liquids to solids, but the main problem is that the process conditions not only lead to the loss of nutrients, bioactive compounds and sensory properties of the food but can also lead to browning reactions, severe dehydration, the formation of hydroxymethylfurfural, furfural, and acrylamide (Moreno-Vilet et al., 2018). For this reason, innovative non-thermal technologies have become increasingly important in recent years, as they can avoid the detrimental effects of thermal processing. They have low energy consumption, are adaptable, can be used on a larger number of food products and show better results in the control of filamentous fungi, spores and mycotoxins compared to thermal technologies (Yousefi et al., 2021), thus ensuring food safety and delivering a high-quality product. Therefore, this review aims to explore novel non-thermal technologies such as high pressure, ozone, UV treatment, blue light treatment, pulsed electric fields (PEF), cold plasma, electron beam (e-beam), plasma jet, pulsed light and nanomaterials that are becoming increasingly popular in food processing. In addition, using novel technologies that are more environmentally friendly, cleaner, and more efficient than conventional methods could help the food industry reduce its environmental impact. In addition, non-thermal technologies offer several potential benefits, such as improving the health attributes of foods by increasing the bioavailability of micronutrients and phytochemicals from both animal and plant sources, which have nutraceutical applications. Moreover, these technologies are used to enhance the physiological properties of proteins and carbohydrates in food, enabling their use in various food processing applications. This includes improving the release of taste compounds and enhancing the sensory quality of fermented products (Gao et al., 2019). However, there are some practical barriers that may arise for the use of non-thermal technologies, such as higher initial investment costs, high operating efficiency and higher processing costs with a higher energy input than a conventional thermal process with heat recovery capacity. In addition, the industrial scale of these technologies is a challenge as laws and regulations need to be put in place to ensure the safety of the processed food (Chacha et al., 2021).

The use of non thermal technologies to reduce fungal and mycotoxin in foods deserves attention, as demonstrated by some other reviews already published in the scientific literature; however, some of them are limited only to detoxification/removal of mycotoxins with the exclusion of fungi inactivation (Adebo et al., 2021; Wang et al., 2023a; Yousefi et al., 2021); or are not specifically focused on fungi (Lopes et al., 2023) or not updated to the last recent developments in innovative technologies (Deng et al., 2020).

This paper provides a comprehensive overview of using specific non-thermal technologies to reduce fungi and mycotoxins during post-harvest processing to maintain product quality. In addition, the opportunities and limitations of newly developed non-thermal methods are explored, providing valuable insight into emerging trends in food processing over the last 6 years. This paper contributes to understanding these innovations and their practical applications in various post-harvest products.

## 2 High-pressure processing (HPP)

High-pressure processing or high hydrostatic pressure (HHP) requires the immersion of a product into water and exposure to high hydrostatic pressure (from 100 up to 800 MPa) in a vessel (Aganovic et al., 2021). The product is commonly packed in a high-pressure-suitable packaging and held under pressure for some minutes until decompression. There are two types of HPP techniques depending on the type of food to be treated. Batch-type HPP is used for solid foods or products with big solid particles, whereas semi-continuous HPP is used for liquid and other products that may be pumped because they have the flexibility to be treated by semi-continuous procedures (Huang et al., 2020).

Although HPP is considered a non-thermal process, the increase in pressure causes adiabatic heating with a temperature rise of 2–3°C/100 MPa for water-based foods and up to 9°C/100 MPa for oil-based foods (Sehrawat et al., 2021). HPP is generally applied for food processing and shelf-life extension due to their inactivating effect on pathogenic and spoilage microorganisms. The determining factors are the types of microbes and their growth phases, duration of processing, pressure, food content, temperature, pH and water activity. Overall, HPP affects only non-covalent bonds as these are sensitive to pressure. HPP may be an effective post-harvest strategy to reduce microbial pathogens in fresh produce if plant tissue damage is minimal.

### 2.1 Mechanisms of action

In general, microorganism cell membrane damage is a critical factor in triggering cell death during HPP treatment and can lead to leakage of intracellular components and loss of homeostasis (Woldemariam and Emire, 2019). These effects lead to impaired cell functions. Four factors contribute to pressure-induced cell death in vegetative microorganisms: (i) unfolding of proteins and enzymes, including partial or complete denaturation; (ii) cell membrane phase transition and change in fluidity; (iii) disintegration of ribosomal subunit; and (iv) changes in intracellular pH related to enzyme inactivation and membrane damage (Gouvea et al., 2023). About fungal spores, HPP also leads to ruptures in the cell wall and membrane and cell membrane of ascospores and the ornamentation on some of these ascospores, which resemble crowns. Recently, Hsiao et al. (2020) reported a degradation of intracellular and extracellular proteins leading to an inactive state in *Aspergillus flavus* spores and the formation of pores in the cell membrane and wall.

Studies showed that HPP could reduce mycotoxins by altering their chemical structure and inducing changes in noncovalent bonds (Azam et al., 2021). To date, however, only few studies have investigated the sensitivity of mycotoxins to HPP treatments. The majority of studies have been conducted using patulin in liquid media. Overall, evidence suggests that thiol content is critical factor for the HHP assisted degradation of patulin. Avsaroglu et al. (2015) reported the degradation of patulin reduction in apple juice with pressure in the range of 300–500 MPa, and attributed this phenomenon to the formation of adducts, with reduced toxicity, between patulin and environemental compopunds with sulphhydryl groups including cysteine and glutathione.

Research is showing that the effectiveness of HPP varies depending on the type of mycotoxin. For example, aflatoxins and ochratoxins are more resistant to HPP, while patulin and zearalenone show higher susceptibility to pressure treatments (Azam et al., 2021).

### 2.2 Filamentous fungal suppression and mycotoxin reduction

It has been demonstrated that vegetative fungal cells are easily inactivated by HPP at low temperatures and moderate pressure (300–400 MPa). In contrast, some fungal spores are more resistant (Sehrawat et al., 2021). The primary determinants of ascospore resistance to HPP are age (normally, older ascospores are known to be more resistant than younger ascospores), pH, water activity (*a_w_*), and food composition (Pinto et al., 2020). The body of research produced results indicating that HPP was effective in inhibiting fungal development overall in grains. In this context, Kalagatur et al. (2018) reported high membrane damage and complete inactivation of *Fusarium graminearum* spores inoculated in peptone water treated with HPP at 380 MPa and 60°C for 30 min. However, in maize grains, the total inactivation of spores was detected at 550 MPa and 45°C for 20 min, indicating that water activity and food matrix are important for the HPP efficacy. In this context, the growth *Fusarium culmorum* in winter wheat submerged in distilled water was suppressed after treatments with HHP 100–300 MPa for 10–600 s, for 6 weeks (Schmidt et al., 2019).

On the other hand, molds were completely inactivated by the HPP treatment (600 MPa for 3 min at 4°C) on pitted sour cherries and they stayed at very low levels for the whole 5 months of refrigerated storage (Tenuta et al., 2023). It is to highlight that the low susceptibility of the aged fungal spores to HPP could be a concern for fruit product manufacturers because spores contaminating fruits can be very old. Therefore since industrial-scale HPP machines can operating at maximum pressures of 650 MPa with or without temperatures up to 50°C, scientific challenges related to the inactivation of resistant spores remain unresolved (Echenique-Martínez et al., 2023). The age-related changes in pressure resistance of ascospores may be due to ongoing changes in the ultrastructural state of the multilayer ascospores wall. Low pH has been proven in some cases to increase reductions while higher pH and media rich in nutrients can improve the spore’s survival; low water activity (*a_w_*) products offer baroprotection, with differing results based on the solute. More recently, an emerging pathway that could be interesting for mold spores’ inactivation is represented by the induced nutrient-like physiological germination that was reported to occur in spores under low/moderate hydrostatic pressures (150–200 MPa), and that was responsible for inactivation of baroresistant microorganisms after germination (Pinto et al., 2024).

Few studies have investigated the sensitivity of mycotoxins to HPP treatments. Kalagatur et al. (2018) found that the level of deoxynivalenol (DON) and zearalenone (ZEA) produced by *F. graminearum* in maize were completely reduced (550 MPa, 45°C for 20 min). On the other hand, Schmidt et al. (2019) found for cereal that HPP (300 MPa, 30°C for 10 min) reduced the concentration of DON, ochratoxin A (OTA), and ochratoxin B (OTB) below detection limits. Additionally, high-pressure treatments effectively degrade other fungal biomolecules that can deteriorate food quality. Although the results are encouraging, the cereal industry still finds it difficult to implement this technique.

### 2.3 Drawbacks

One of HPP application’s primary limitations in reducing fungi and mycotoxins is its reduced efficacy against dry foods and products with a water activity below 0.8. Additionally, the thick cell walls and dehydrated spore core of fungal spores render them highly resistant to HPP inactivation. High-pressure processing requires a very high level of initial investment and a relatively high maintenance cost (Cacace et al., 2020), which includes energy consumption, equipment maintenance, and the cost of water used to generate the pressure, which can limit its accessibility for small and medium-sized companies. Integrating HPP equipment into existing production lines may require significant modifications. The equipment works only in batch or semi-continuous mode, implying additional costs and downtime. HPP equipment has a limited size and capacity, which can restrict its use for high-volume or irregularly shaped products.

## 3 Ozone (O_3_) treatment

Ozone treatment is considered a safe method for food because it decomposes into oxygen and does not form residues that affect consumers’ health. The most common gaseous ozone generators work through Corona Discharge (CD), with 600 to 2 kHz frequencies. Ozone in the aqueous phase (ozonated water) can be used in fresh foods; however, its effect on fungal inactivation is lower due to its low stability in this medium (Davies et al., 2021).

### 3.1 Mechanisms of action

Ozone can diffuse through the cell wall of filamentous fungi, reach the cytoplasm, and change cell activity (Deng et al., 2020). In the fungal cell envelope, polyunsaturated fatty acids are affected by ozone, whereby the membrane permeability enables electrolytes and the contents of cells to leak out (Ouf and Ali, 2021). One aspect of ozone toxicity is its ability to form reactive oxygen species (ROS), which oxidatively destroy biological components, causing cellular dysfunction or cell death. Additionally, ozone inhibits the expression of genes involved in ergosterol synthesis (Li et al., 2022), and decreases the quantity of −1,3-glucan rather than chitin in the inner layer of the cell wall (Ali and Abdallah, 2024); in addition O_3_ oxidizes sulfhydryl and amino acid groups of enzymes, resulting in a reduction of spore development and germination and causing rapid cell death (Afsah-Hejri et al., 2020). Fungal spores differ in sensitivity to ozone, and it seems to be directly linked to spore surface (Pagès et al., 2020) and to differences in their component content, which might accelerate or decrease the toxic action of ozone (Ali and Abdallah, 2024).

It is known that the functional groups inside the mycotoxin molecules would be able to interact with the oxidizing agents. It may change their chemical structures, allowing for the creation of products with fewer double bonds, a lower molecular weight, and lower toxicity. Afsah-Hejri et al. (2020) provided a detailed review of the recognized studies on the mechanisms of mycotoxin degradation by O_3_: (i) O_3_ reacts with nitrogen heterocycles of fumonisins (F) and forms N-oxide, the primary amine of the molecule; (ii) in the case of ochratoxin A (OTA), O_3_ attacks the chlorinated ring structure resulting in amino acids or free chlorine; (iii) in the deoxynivalenol (DON), O_3_ attack the C9–C10 double and oxidizes the allylic carbon in the C8; (iv) ozone attacks on the furan ring of the aflatoxin C8-C9 double bond degrading their structure; (v) patulin tend to be more degraded than complex mycotoxins due to its simple structure producing diglycolic acid, oxalic acid, and CO_2_ as final products.

### 3.2 Filamentous fungal suppression and mycotoxin reduction

Research conducted in maize demonstrated the reduction of *Aspergillus* spp. and *Penicillium* spp. when treated with of 2.14 mg/L of the gas up to 50 h (Brito et al., 2018), and 13.5 mg/L for 24 h and 36 h (Ribeiro et al., 2022). The results show that in *Aspergillus* species, the pigmentation is deeper, and their conidia are comparatively thick-walled, which could provide protection against the sun’s ultraviolet radiation and possibly against gases such as O_3_. In addition, physiology, morphology among genera, moisture content, and substrate variations may influence the sensitivity or resistance of fungal spores to O_3_ (Akbar et al., 2020; Conte et al., 2020). In this context, in coffee beans infected with *Aspergillus westerdijkiae*, *A. ochraceus*, and *A. carbonarius*, treatments with 600 mg/L O_3_ at low water activity (*a_w_* = 0.75) reduced significantly fungal populations and therefore the mycotoxin content during the storage (12 days). However, when *a_w_* was maintained at values of 0.90 and 0.95, toxigenic fungal populations increased dramatically 48 h after exposure (Akbar et al., 2020). It should be borne in mind that in many products with low water activity, molds are present in the form of spores, which, as already mentioned, are more resistant to ozone. The inhibitory effect of ozone on spore formation has considerable commercial potential, as the treatment breaks the infection cycle. However, the effectiveness of ozone under *in vitro* conditions is not able to reduce fungal growth. It is, therefore, important to optimize the composition or treatment of ozone.

Table 1 depicts the most significant reports, in the last years, of ozone fumigation on postharvest pathogenic fungi in seeds, cereals, and fruits. A significant part of the works demonstrated that O_3_ treatment had a clear inhibitory effect on molds in a dose- and time-dependent manner, potentially preventing product decay.

**Table 1:** Summary of recent studies on filamentous fungi reduction by ozone.

Substrate	Process parameters	Observations	References
Brazil nuts	O_3_ concentration: 2.42, 4.38, 8.88, and 13.24 mg O_3_/L. Time: 0, 60, 120, 180, and 240 min. Flow rate of 3.0 L/min.	CFU count of *A. flavus* decreased from 5.34 to 2.21 Log CFU/g at 8.88 mg O_3_/L for 240 min.	de Oliveira et al. (2020)
Wheat seeds	O_3_ concentration: 2000 mg O_3_/h. Time: 15, 30, and 45 min. Temperature: 30, 40, and 50°C of drying air temperature.	The maximum reduction of fungal count was 92.86%, with reduction from 1.87 CFU/g to 0.13 CFU/g, when the wheat seeds was treated with ozone for 45 min and dried with air temperature at 50°C.	Granella et al. (2018)
Rice storage	O_3_ concentration: 0.393 kg O_3_/m^3^. Time: 30, 90, and 180 min.	Ozonation for 180 min caused 90% fungal elimination, and some strains remained resistant.	Savi et al. (2020)
Peanuts	O_3_ concentration: 0.94, 1.89, 2.83, and 3.78 mol O_3_ /kg peanut Airflow rate: 0.19 mol/min.	The higher exposure (240 min = 3.78 mol O_3_/kg peanut) reached 92% of fungal growth inactivation, including aflatoxigenic fungi. A minimum ozone dosage to eliminate fungi and not cause lipid peroxidation on the peanut surface.	Gomes et al. (2023)
Winter jujube (*Ziziphus jujuba* Miller cv. Dongzao)	O_3_: 0, 2.5, 5 and 10 μL/L, 1 h per each day of storage, temperature of storage: 25 ± 0.5°C	The growth of fungi was largely inhibited by ozone, with concentrations being higher than 2.5 μL/L, indicating that ozone could reduce the fruit decay of jujube by inhibiting fungal proliferation on the fruit surface.	Zhang et al. (2022)
Cantaloupe	O_3_: 0, 6.432, 10.720, and 15.008 mg/m^3^ for 1 h. The ozone treatment occurred weekly in a fumigation device with a storage capacity of 1,200 L.	Reduction in the relative content of *Alternaria* spp. due to the increase in ozone concentration.	Chen et al. (2020)
Muskmelon (*Cucumis melo* L.)	O_3_: 1.10 and 2.20 mg/L per 30, 60, and 120 min.	The development of Fusarium rot was effectively controlled in muskmelon inoculated with *Fusarium sulphureum* after ozone treatment. The concentrations of NEO in the rotten part with ozone treatment after 30, 60, and 120 min were 0.84, 0.17, and 0.14 times lower than those in the control for 8 days of storage.	Hua-Li et al. (2018)
Mango (*Mangifera indica*) fruit	O_3_: 0.25 mg/L for 24 or 36 h at 10°C. Experimental fruit were stored at 90% relative humidity and 10°C for three weeks and ripened at ambient temperature for 1 week.	Ozone treatment (24 h) decreased mycelial growth by 60.35%. On day 21, untreated fruit had a high disease incidence *Lasiodiplodia theobromae* (68.33%) compared to O_3_ (24 h) (53.29%) and O_3_ (36 h) (43.30%).	Bambalele et al. (2023)
Papaya (*Carica papaya*) fruit	Ozonated water (3 mg/L) at 20 ± 2°C for 300 s.	Reduced the stem-end rot, controlling around 50% of the severity, and delaying the onset of the symptoms in 3 and 4 days. The integrated approach combining heat treatment, by immersing the papaya peduncle in hot water at 70°C for 15 s, followed by immersion in ozonated water (3 mg/L) for 300 s, efficiently controlled the stem-end rot (>90%).	Terao et al. (2019)
Blueberry fruit (*Vaccinium corymbosum*, cv. O’Neal)	O_3_: 18 mg O_3_/L for 10 and 20 min. Exposure to 18 mg O_3_/L for 10 min	Slight but no significant differences for native mycobiota and *Botrytis cinerea* incidence reduced the percentage of infected fruit by ~34 and 40%.	Jaramillo-Sánchez et al. (2019)

On the other hand, several studies have shown that the O_3_ efficacy in decontaminating mycotoxins depends on their concentration, exposure time, ozone state, matrix moisture content, weight, and specific surface area. The treatment efficiency increases with temperature, with increased ozone exposure (higher ozone concentration or longer treatment times), and better ozone diffusion within the matrix. For example, in naturally contaminated ground corn (10% moisture content), treatments of 40 mg/L of O_3_ led to a reduction of up to 68.1% of ZEN in 120 min while the 70.3% of OTA reduction required 180 min; on the contrary, DON appeared to be quite difficult to be degraded (Krstović et al., 2021). In contrast, Purar et al. (2022) reported the reduction of DON in naturally contaminated maize to levels below the detection limits (40, 70, and 85 mg/L for 180 min), and differences could be related to the mycotoxin concentration. The ozone efficacy has also been demonstrated in the reduction of aflatoxin B_1_ (AFB_1_) and aflatoxin G_1_ (AFG_1_) naturally present in corn grit (9.5% of moisture content), as well as ZEN and AFs content in both naturally and artificially contaminated ground maize with a degradation level that was dependent on ozone exposure (Purar et al., 2022). In wheat bran, ZEN was degraded by a major percentage with respect to DON (Santos Alexandre et al., 2018). Also significant reduction of AFB_1_ and AFG_1_ was reported in pistachios and almonds (Ali and Abdallah, 2024; Babaee et al., 2022). On the other hand, Ozone treatment reduced the accumulation of diacetoxyscirpenol (DAS) by *Fusarium sulphureum* inoculated in potato tuber by down-regulating expression of genes involved in the DAS biosynthesis pathway (Li et al., 2022).

### 3.3 Drawbacks

Ozone is an unstable gas and decomposes rapidly, so specialized equipment is required to generate it *in situ*, which increases investment and operating costs. In addition, the adaptation of existing production lines, the corrosiveness of ozone towards certain materials, and the health risks for operators require rigorous safety and control measures. Accurate ozone dosing is critical to prevent adverse product and environment effects. Excessive ozone exposure can lead to the degradation of proteins, vitamins, and lipids, resulting in undesirable compounds and altered sensory properties. Moreover, ozone’s limited selectivity can generate undesirable by-products, underscoring the importance of precise dosing and control in ozone applications. On the other hand, batch or semi-continuous operation limits the scalability of ozonation processes or limits them to bulk product storage operations.

## 4 UV treatment

Ultraviolet refers to electromagnetic radiation with a wavelength shorter than visible violet light and is categorized according to its energy level: UVA (320–400 nm), UVB (280–320 nm), and UVC (200–280 nm). The dose of UV radiation given determines how effectively it disinfects. It is defined as the product of intensity (mW/cm^2^) and the exposure time (s) and is commonly expressed as mWs/cm^2^ or mJ/cm^2^.

### 4.1 Mechanisms of action

UV radiation can denature microorganisms’ DNA, proteins, or lipids, causing death or inactivation. When nucleotide bases are exposed to UV light, pyrimidine (6–4) pyrimidone photoproduct [(6–4) PP] and cyclobutene pyrimidine dimer (CPDs) are formed. These may cause mutations and alter patterns of gene expression and cell death. A smaller amount of UV light is also absorbed by other cellular constituents, such as aromatic amino acids, which results in photo-fragmentation, loss of enzyme specificity, and other biological effects (Wong et al., 2019). The wavelength of the incoming photons, the DNA sequence, the flexibility of the DNA structure, and the base locations all have a significant impact on the type and quantity of lesions that occurs. On the other hand, other indirect damages are induced by UV treatment, such as water radiolysis, which can lead to the accumulation of ROS within cells. In addition, UV radiation can also degrade mycotoxins, it is commonly recognized that many mycotoxins with complex structures and more reactive functional groups can absorb UV light, which can trigger photoreaction and reduce mycotoxin.

### 4.2 Filamentous fungal suppression and mycotoxin reduction

According to Esua et al. (2019), the mechanism of action of UV-C radiation in strawberries, tomatoes, mushrooms, and grapes is related to the activation of regulatory enzymes for the production of phenolic compounds with antifungal properties. Major studies reported that the effect of UV radiation on conidia is easier compared to mycelium. In fact, fungal mycelium has a higher metabolic activity than conidia, which are largely dormant or inactive structures with high resistance against various stresses (Baltussen et al., 2020). Several authors reported the efficacy of UV radiation treatment by inducing DNA damage conidia, preventing its germination and multiplication, nevertheless, the effect is fungal species-dependent, and the spore resistance could be due to the thicker conidial wall. In this context, Nakpan et al. (2019) reported that UV treatment for 10 min against *A. fumigatus* was ineffective on conidia due to their defense mechanisms correlated with melanin, thus suggesting that the exposure to ultraviolet radiation must be more significant to destroy the cellular structure of *Aspergillus* conidia substantially. Simultaneously, other authors reported that *A. fumigatus* and *A. niger* conidia are highly resistant due to the involvement of DHN-melanin against UV-C treatments (Blachowicz et al., 2020; Cortesão et al., 2020).

*In situ* studies revealed good efficacy of UV treatments to reduce the disease severity (near 90%) of *B. cinerea* in mature strawberry fruits (1.3 mJ/cm^2^ UV-B treatment) (Abd El Hamid et al., 2022), as well as the reduction of the total fungal colonies in the brown, black, and red rice treated with UV-C for 3 h (Ferreira et al., 2021) and the growth of *Penicillium* spp. (79% reduction) and *Fusarium* spp. (62% reduction), following treatment of maize kernels with a UV-C dose of 5,000 mJ/cm^2^ (Popović et al., 2018).

UV irradiation has been shown to be an effective method of destroying mycotoxins in many agricultural products. The studies reported either the inactivation of mycotoxins directly and by means of the inhibition of the fungal mycotoxin producer, in this context, the efficacy of UV-C irradiation to completely degrade AFB_1_, OTA, and aflatoxin B_2_ (FB_2_), but not against nivalenol (NIV) on semolina has been reported (Shanakhat et al., 2019). Also, a low dose (25 mJ/cm^2^) of UV-C treatment inhibited the production of Alternariol (AOH), alternariol monomethyl-ether (AME), and tenuazonic acid (TeA) in tomato fruits inoculated with *Alternaria alternata* (Jiang et al., 2019); however a reduction of 70% of patulin in apples inoculated with *P. expansum* required a higher dose (3,510 mJ/cm^2^) (Nicolau-Lapeña et al., 2023). Aflatoxins (B_1_ + B_2_ + G_1_ + G_2_), DON, OTA, and ZEN were reduced in the brown, black, and red rice after UV-C treatment for 1 and 3 h when compared to freshly-harvested grains and those without UV-C treatment (Ferreira et al., 2021). Furthermore, Popović et al. (2018) determined that postharvest UV-C treatment of corn kernels by using 253.7 nm sources was efficacious in reducing DON (30%), OTA (17%) and ZEN (52%) accumulation.

### 4.3 Drawbacks

Despite its effectiveness, UV disinfection has limitations inherent to the nature of UV radiation. The UV penetration power is insufficient to completely destroy microorganisms when the initial total microbial count is high (Deng et al., 2020). Limited penetration in turbid media, intensity attenuation, and shadow formation restrict its application in heterogeneous systems, thus requiring higher doses or recirculation systems, which increase operating costs. In addition, UV-induced photochemical reactions can generate undesirable by-products that may be toxic or have bad taste and odor (Byun et al., 2020). Industrial implementation requires careful design of reactors, considering factors such as UV dose and energy efficiency. Energy costs associated with operating UV lamps can be high. Strict safety measures, such as physical barriers, interlock systems, and personal protective equipment, must be implemented to protect against the harmful effects that UV radiation can cause to the skin and eyes of operators.

## 5 Blue light (BL) treatment

The antifungal properties of light are a research area that is gaining more attention due, in part, to the development of resistance to standard control methods. Several researchers have evidenced that among different visible light wavelengths (blue, green, and red light), only the blue spectral range from 400 to 470 nm allowed for detectable antimicrobial effects (Leanse et al., 2022). Alternatively, a low-cost, environmentally friendly, non-thermal sanitization system may be created using light-based technology, specifically light-emitting diodes (LED); it is a semiconductor material doped with impurities that create a boundary or interface (known as a p-n junction) between two types of semiconductor materials, one type (the positive or p-type) having an excess of holes and the other type (the negative or n-type) having an excess of electrons. LEDs function according to the electroluminescence principle, which states that they emit light when an electric or magnetic field is applied. Excited electrons release energy in the form of electromagnetic radiation and produce light when they move toward lower energy states in an electric or magnetic field.

Many microorganisms are sensitive to BL, which can lead to physiological reactions triggered by blue light receptors. In particular, many fungal species have their wavelength receptors, and all of these receptors have an organic compound of low molecular weight, such as flavin, retinal, or tetrapyrroles, for the perception of blue, green, and red light, respectively (Leanse et al., 2022). When fungal cells are exposed to BL, light-sensitive molecules within the cells absorb the light. This absorption stimulates the chromophores, which leads to the production of ROS. Moreover, another proposed mechanism of inactivation for several microorganisms is represented by the natural accumulation of photoactive metal-free porphyrins such as uroporphyrin, coproporphyrin, and to a lesser extent protoporphyrin due to exposure to blue light (Dong et al., 2022).

### 5.1 Mechanisms of action

The widely accepted mechanism by which blue light (BL) induces fungal cell death involves the photoexcitation of photosensitizing chromophores, such as iron-free porphyrins, flavins, and key proteins like White Collar WC-1, WC-2, and cryptochromes. This process generates intracellular reactive oxygen species (ROS), which compromise cell membrane integrity, damage DNA, trigger lipid peroxidation, and potentially destroy mitochondrial membranes. As a result, mitochondrial dysfunction occurs (Zhu et al., 2023), leading to extensive cellular damage and ultimately cell death through necrosis. Additionally, research by Grangeteau et al. (2018) has shown that BL disrupts the spore cell wall and membrane, reducing spore viability and further contributing to fungal cell death. The proposed mechanism behind the action of blue light is the photoexcitation of endogenous porphyrins, resulting in ROS, loss of cell membrane integrity, and cell death.

Although BL treatment demonstrated a lethal effect for some filamentous fungi, its activity depends on the wavelength, dose, and fungal genus. The studies all clearly agree that BL’s shorter wavelengths (e.g., 400–410 nm) had greater microbicidal effects than the others (e.g., 450–470 nm). These results, when applied after spore germination, are crucial for identifying the best BL wavelength for cleanliness or lowering contaminations. On the other hand, the sensibility of *Aspergillus oryzae* to BL has been linked to the presence of a BL receptor that senses blue light irradiation; in this case, the signal is transmitted to the central regulatory module associated with spores (such as the BrlA→AbaA→WetA regulatory cascade, explained below), which likely led to downregulation of the associated genes and ultimately led to a significant reduction in the number of conidia produced (Shangfei et al., 2021). AbaA is a crucial element for the correct differentiation and function of phialides. It is activated by BrlA during the middle stages of conidiophore development, following the differentiation of the metulae. The *brlA* gene in *Aspergillus nidulans* facilitate the transition from the indeterminate apical growth pattern of the conidiophores. Additionally, the *wetA* gene is induced by AbaA in the later stages during of conidiation and is essential for synthesizing the cell wall layers that enable conidia to mature and become impermeable.

### 5.2 Filamentous fungal suppression and mycotoxin reduction

The efficiency of BL inactivation depends on several variables, including the wavelength of the light, the duration of exposure, the fungal species, and the temperature. Fungal spores are more resistant to environmental stress than vegetative cells, but BL can still impact them. For example, exposure to BL low-intensity suppressed by 81% the development of *Penicillium italicum* spores in mandarin fruits (Bhatta, 2022), controlled *Penicillium expansum* in apples after harvest and affected the expression levels of the virulence genes and, in particular the gene *LaeA* which regulates several secondary metabolite genes, including the patulin gene cluster (Zhu et al., 2023). In addition, it inhibited the growth of *Geotrichum citri-aurantii*, which is responsible for acid rot in citrus fruit, and the mycelial diameter was greatly reduced with increasing BL intensity (Du et al., 2023b).

Blue light can also interfere with the spore germination process, as Trzaska et al. (2017) reported that a 60 min treatment with BL (405 nm) led to a significant inhibition of germination of *Fusarium oxysporum*, *Fusarium solani, Scedosporium apiospermum*, and *Scedosporium prolificans* fungal conidia. In contrast, *Rhizopus microsporus* and *Mucor circinelloides* conidia showed the highest resistance. Other authors reported that a treatment duration of 1 and 12 days under BL led to a drastic inhibition of conidia germination of *A. alternata* (Igbalajobi et al., 2019).

The efficacy of BL at room temperature rather than at lower temperatures (4–16°C) to inactivate fungi was reported in the growth of *B. cinerea* and *Rhizopus stolonifera* on tomatoes and strawberries (Ghate et al., 2021), and in *Geotrichum candidum* and *Fusarium* sp. isolated from litchi fruit (Yu et al., 2023). It is likely that the fungi may have a greater metabolic load at room temperature, which could accelerate the rate of cytotoxic response in addition to a greater production of intracellular ROS formation, which in turn accelerates microbial inactivation. The surface texture of food also plays a role in the effectiveness of BL, as light interacts better with smooth surfaces. However, rough surface textures may allow better harboring or shading of fungal spores in surface defects or cracks as demonstrated by the growth of *Geotrichum candidum* on smooth and rough surfaces of litchi fruit (Yu et al., 2023). More recently, Du et al. (2023a) found that blue light not only directly inhibit the mycelium growth of *G. citri-aurantii* in citrus fruit but also induced the activities of defense-related enzymes and antioxidant enzymes in citrus to limiting the development of sour rot caused by this species.

The reduction of mycotoxin formation is critical for protecting human and animal health, maintaining food quality, avoiding economic losses, and promoting sustainability. Blue light was reported as an effective strategy to control mycotoxins. Zhang et al. (2021) reported that OTA production decreased by 66.88%, when *Aspergillus carbonarius* was grown under blue light. Depending on the fungal species or strain, there are differences in the interaction between light irradiation and mycotoxin production. For example, using BL treatments (455–470 nm), OTA production was reduced by controlling the expression of ochratoxin polyketide synthase in *P. expansum*, *Penicillium verrucosum*, and *Penicillium nordicum* (Zhu et al., 2023). Similarly, BL with a wavelength of 455/470 nm alone, in combination with low a_w_ values (0.95), leads to a significant reduction in AFs and cyclopiazonic acid formation. In addition, reduced transcriptional activity of genes responsible for mycotoxin biosynthesis of *A. flavus* and *A. parasiticus* was observed (Priesterjahn et al., 2020). Similarly, the expression of the genes PatA, PatE, and PatN related to the patulin production decreased by 90, 50, and 72%, respectively, when the culture of *P. expansum* was exposed to 500 μmol/m^2^s^1^ blue LED light treatment (Zhu et al., 2023).

### 5.3 Drawbacks

Among the most widely used blue light sources in industrial applications are LEDs and laser diodes, which are notable for their high efficiency, low cost, and ease of scaling. Femtosecond lasers, while offering exceptional precision, are more suitable for niche applications due to their complexity and cost. Implementing blue light-based disinfection systems can be limited by factors such as light absorption by plastic materials, heat generation, and the need for specialized equipment. In addition, prolonged exposure to blue light can cause eye damage in workers. The effectiveness of this technology depends on variables such as the power of the source, the distance to the surface to be treated, and the presence of biofilms (Hadi et al., 2020). Successful implementation of blue light technology in the food industry requires a comprehensive evaluation of each specific application, considering factors such as the target microorganisms, surface materials, and environmental conditions. While light-based treatments (LB) are generally considered to have a low risk of resistance development due to their multi-targeted nature, there remains a possibility of surviving cells developing tolerance. If the inflicted damage is insufficient to eliminate all targeted cells, those that survive may adapt and become resistant to future treatments (Rapacka-Zdonczyk et al., 2019).

## 6 Pulsed light (PL)

A pulsed light system (PL) is also known as pulsed UV light, high-intensity broad-spectrum pulsed light, pulsed white light, or intense light pulses (John and Ramaswamy, 2018). One of the key advantages of pulsed light (PL) over other non-thermal technologies is its emission of highly concentrated, short-duration light bursts. These intense energy pulses can inactivate microorganisms within milliseconds to seconds, making PL a highly time-efficient method for surface treatments. Its broad-spectrum light, which includes UV, visible, and infrared wavelengths, enhances its ability to target and eliminate a wide range of pathogens effectively (Santamera et al., 2020). A typical PL system consists of a high-voltage power supply, a storage capacitor, a pulse-forming network that determines the pulse shape and spectrum characteristics, a gas-discharge flash lamp, and a trigger that initiates the discharging of the electrical energy to the flash lamp. Electrical pulses are applied to excite inert gases, such as xenon in flashlamps, and cause gaseous molecules to collide, producing light pulses. After that, the light energy is emitted in extremely concentrated short-duration light bursts (lasting for a few hundred microseconds, usually 1 to 1,000 μs). The resulting light has an electromagnetic spectrum ranging from ultraviolet (UV) to near-infrared (IR). This range of electromagnetic radiation includes UV rays (*λ* = 100–400 nm), visible light (λ = 400–700 nm), and infrared (λ = 700–1,100 nm). In order to satisfy the unique process requirements, the PL system can deliver light as a single pulse, a burst of pulses (timed mode) at a frequency of 1–20 Hz with a pulse width of 300 ns to 1 ms, or a continuous array of pulses in random sequences. Optical sensors measure the fluence of the PL irradiation on the sample. Numerous variables, such as the number of flashes, the pulsed energy level, the distance between the sample and the lamps, and the type of sample treated, affect the inhibitory effect of PL (Rifna et al., 2019).

### 6.1 Mechanisms of action

The effects of PL on microbial cells are divided into three categories: (i) The photochemical effect, caused by PL UV component that can be absorbed by DNA and other cell components, refers to the water vapor-induced damages like disruption in the cell wall, shrinkage in the cytoplasmic membrane, and mesosome rupture, followed by leakage of cell content and genetic material (Contigiani et al., 2021), the absorption of UV fraction initiates the DNA or RNA damage by forming pyrimidine dimers, resulting in mutations, inhibition of DNA, and thus prevents the microorganisms’ ability to replicate; (ii) the photothermal effect caused by the visible and near-infrared regions, which only generate heat high enough to kill microorganisms on the surface of the treated substrate (a few μm thick), and (iii) the photophysical effect caused by the high-power pulsing effect, which is constantly disturbing structure damages.

Many factors influence microbial inactivation, such as (i) food surface, in this case, non-uniform exposure of the sample reducing the inactivation efficiency; (ii) food shape, spherical shape is the most suitable shape; (iii) distance of exposure of light (iv) food color media, (v) degree of heat dissipation and its absorption by the food matrix, and (vi) turbidity that may be caused by the presence of particles that have a high UV absorbance, which could lower overall PL efficiency. In addition, after PL treatment, temperature, moisture content, and lighting are considered significant environmental variables that can influence microbial activation and inhibition. However, the intrinsic properties of the microbial cells—that is, the kind of microorganism, the growth stage, and the size of the inoculum—also influence PL lethality. For instance, it is commonly known that microorganisms’ PL susceptibility follows the following pattern: Gram-negative bacteria < Gram-positive bacteria < yeasts < bacterial spores < molds < viruses. Recently, transcriptomic analysis on *A. carbonarius* treated with fluences of 9 J/cm^2^ (600 s) conducted by Wang et al. (2023b) revealed that the mechanisms of mold suppression are caused by down-regulation of differentially expressed genes related to energy and glucose metabolism, transport, stress response, and secondary metabolism in addition to inhibition of DNA replication and disruption of the cell wall and cell membrane. Transcriptomic analysis of pulsed light inhibition of *Aspergillus carbonarius* growth detected negative effects on DNA replication, glucose metabolism, cell integrity, and secondary metabolism (Wang et al., 2023b). Mycotoxin destruction should be attributed to photochemical mechanisms, which in this case include mycotoxin fragmentation, disruption of the terminal furan double bond, and lactone ring opening (Abuagela et al., 2018).

### 6.2 Filamentous fungal suppression and mycotoxin reduction

Research on fresh fruits, including melons and strawberries, demonstrated that PL only reduces lesions’ size and decay rate. Specifically, the inhibition of *Fusarium pallidoroseum* growth in melons (*Cucumis melo* var. Spanish) treated with a dose of pulsed light (PL) of 9 J/cm^2^ (0.3 μs), was linked to the upregulation of some fruit biomarkers, including pipecolic acid, saponarin, and orientin, which function as key indicators of the fruit’s defense mechanism against pathogens (Filho et al., 2020). Romero Bernal et al. (2019) observed that in strawberries artificially contaminated with *B. cinerea*, and there was a 2-day delay in the infection’s onset and a lower mold incidence when treated with PL for 1 to 40 s (fluences: 1.2 to 4.78 J/cm^2^), respectively; compared to the control group in 10 days of cold storage. On the contrary, low efficacy of PL (fluence: 1.9 J/cm^2^) was observed in fungal spoilage of strawberries (cv. Albion) stored at 5°C (Contigiani et al., 2021).

It is well recognized that PL technology is more suitable for surface decontamination in foods with smooth surfaces, such as fresh whole fruit and vegetable commodities than in cereals, as demonstrated by Zenklusen et al. (2018) on the inactivation of *Aspergillus carbonarius* and *A. flavus* in malting barley. In this case, the authors found that even after extended treatment times, there was a persistent residual population, and a reduction of 1.2–1.7 log cycles up to 5–15 s (fluence: 6.0–18.0 J/cm^2^) was recorded. The noticeable tailing observed in both molds could be attributed to either the inoculated conidia not receiving a uniform treatment due to one side of the grain being exposed to radiation or the uneven distribution of conidia on the grain surface. The restricted penetration of PL into food, on the other hand, is regarded as a shortcoming of this technology.

The impact of PL treatment on mycotoxin decontamination *in vitro* and on fruits has been the subject of numerous studies. Table 2 summarizes the percentage decrease by light pulses of the most well-known mycotoxins reported in recent years. The studies found that variations in mycotoxins’ degradation capacity are caused by differences in molecular structures, which alter photodegradation efficiency.

**Table 2:** Percentage reduction of the best known mycotoxins by treatment with Pulsed Light.

Mycotoxins	Substrate	PL conditions	Decrease (%)	References
DON	Germinating barley	PL intensity 0.528 J/cm^2^/pulse (fluence = 1.56 J/cm^2^ for 60 s)	35.5	Chen et al. (2019)
OTA	Grape juice	PL intensity of 0.98 J/cm^2^/pulse (fluence = 39 J/cm^2^).	95.29	Wang et al. (2022)
AFB1	Rough rice	PL at intensity of 0.52 J/cm^2^ /pulse (fluence = 84.4 J/cm^2^ for 80 s)	75	Wang et al. (2016)
AFB2			39	
AFB1	Rice bran	PL at intensity of 0.52 J/cm^2^ /pulse (fluence = 16.1 J/cm^2^ for 15 s)	90.3	
AFB2			86.7	
AFB1	Red pepper powder	PL intensity 0.15 J/cm^2^/pulse (fluence = 1.0 to 9.1 J/cm^2^ for 20 s)	67.2	Woldemariam et al. (2022)
Total aflatoxins (AF)			50.9	
(OTA)			36.9	

AFB: Aflatoxin B_1_ and B_2_; DON: Deoxynivalenol; OTA: Ochratoxin A.

### 6.3 Drawbacks

One of the technique’s key limitations is its limited penetrating power; hence, opacity, topography, and matrix composition all significantly impact process effectiveness. If the treated surface has a rough texture or a pored structure, a shadow effect occurs, allowing microorganisms to survive the treatment. The large-scale implementation of PL systems in production environments presents significant challenges. The effectiveness of PL depends on several factors, such as the wavelength of the emitted light (generally between 200 and 300 nm for maximum germicidal action), the fluence (energy per unit area), and the pulse duration. These parameters must be carefully adjusted for each application since excessive fluence can damage materials, and insufficient fluence may not guarantee the complete inactivation of microorganisms. In addition, the generation of ozone as a byproduct of the interaction of UV light with atmospheric oxygen can sometimes limit its application. The costs associated with the acquisition and operation of PL equipment and the need for trained personnel to operate it constitute another obstacle to its widespread adoption. Despite these challenges, ongoing research in the field of PL seeks to optimize this technology, develop more efficient and compact light sources, and explore new applications in the food industry, such as packaging treatment and wastewater decontamination.

## 7 Pulsed electric fields (PEF)

PEF technology uses strong electric field pulses with durations ranging from a few microseconds to milliseconds and intensities ranging from 10 to 80 kV/cm. The product is positioned between two electrodes, and the number of pulses applied determines its efficacy. Numerous ways, including bipolar waves, exponentially decaying waves, and oscillatory pulses, can be used to apply the electric field (Abbas Syed, 2017).

### 7.1 Mechanisms of action

The inactivation of microorganisms occurs by electroporation or permeabilization of membranes caused by the electric field, which allows the movement of extracellular and intracellular molecules. For electroporation to occur, the electric field must be above the threshold value at which irreversible rupture of the cell membrane occurs, leading to loss of microbial homeostasis and, ultimately, cell death (Kranjc and Miklavčič, 2017). This threshold value, or critical electric field strength, is different for each microorganism. However, it is generally at 15–35 kV/cm. Several parameters can influence specific microorganism inactivation during PEF processing. These key parameters include field strength, treatment time, temperature, pulse shape, microbe type, development stage, and treatment substrate properties.

In filamentous fungi, field intensity, number of pulses, and pulse duration and number of hyphae, thickness, size, and shape of the cell membrane are the most important critical factors (Feng et al., 2019). A theoretical study with a cell dielectric model in filamentous fungi found that the optimal pulsed electric field parameters for inactivation are an electric field of 13 kV/cm and a pulse duration of 1,000 ns (Feng et al., 2019).

### 7.2 Filamentous fungal suppression and mycotoxin reduction

There are few reports of the application of this technology on fungal suppression. Most of the research has been registered in the treatment of juices or liquid products where the incidence of fungi is lower. Some significant advances have been made in applying PEF on food-related fungi. For example, PEF has been investigated on wheat seeds, where it improved vigor and germination but failed to suppress the fungi completely. A maximum reduction of 2.85 log CFU/mL (12 kV/cm) has been reported, while other conditions reduce surface fungi and yeasts by only 1 log CFU/mL (Evrendilek et al., 2021). In sesame seeds, 56.1% of the inactivation of *A. parasiticus* was obtained after PEF treatment using 17.28 J of energy (19.79 s × 180 Hz) (Bulut et al., 2020).

The efficacy of PEF against aflatoxin naturally present in red pepper flakes was studied (Akdemir Evrendilek et al., 2022): a reduction of nearly 98% was obtained in samples containing low quantities (from 14.88 to 43.02 μg/L) of several aflatoxins, while the reduction was lower in samples containing high quantities. Similar results were observed in sesame seeds (Bulut et al., 2020). This reduction could be due to adding OH^−^ and H^+^ reaction groups to double bonds of AFB_2_ structure and the loss of methylene group (Pallarés et al., 2021). Very recently, Stranska et al. (2023) demonstrated the potential of PEF to reduce mycotoxins content in cereals via the generation of different reaction products from the parental chemicals and through improved matrix extractability into conductor water. Based on the dry matter of barley, the authors found that PEF-induced reductions in trichothecenes, ZEN, enniatins, beauvericin (BEA), and tentoxin were up to 31, 48, 84, 36, and 46%, respectively. While reductions were also noted for most mycotoxins in water, an increase was noted for BEA, ZEN, enniatins, and type A trichothecenes—likely due to easier extraction from the matrix. Hydrolysis, elimination, and/or oxidation are primarily responsible for the breakdown and transformation of mycotoxins. The authors demonstrated the existence of ester bond hydrolytic products, specifically for enniatins. They suggested that oxidation broke the bond between the amino acids *β*-carbon and its *α*-functionalized carboxylic group.

### 7.3 Drawbacks

The fundamental disadvantage of PEF technology is that it is only suitable for particles suspended in liquids. Furthermore, the presence of bubbles during treatment and the heterogeneity of foods, especially those with high concentrations of solids or particles, can hinder the uniform distribution of the electric field, leading to operational problems and non-uniform treatment. The large-scale implementation of this technology presents considerable challenges. The effectiveness of PEF depends on several factors, such as the electrical conductivity of the food, the geometry of the treatment chamber, and the frequency of the pulses. In addition, the high costs of PEF equipment, including power supplies, control systems, and specialized electrodes, represent a significant barrier to its widespread adoption in the food industry. To overcome the limitations of PEF technology, researchers are actively exploring innovative solutions, such as developing novel electrode materials, improving control systems, and integrating PEF with other food processing techniques.

## 8 Cold atmospheric plasma (CAP) treatment

Plasma is a collection of various excited atomic, molecular, ionic, and radical species that coexist with a variety of other particles, such as electrons, ions, free radicals, reactive oxygen/nitrogen species (RONS), gas atoms, molecules in ground or excited state, and electromagnetic radiation (UV photons and visible light) which have a potent oxidizing effect (Laurita et al., 2021; Misra et al., 2019). The production of a variety of components is necessary for the antifungal activity and mycotoxin degradation process, including ultraviolet radiation, reactive oxygen species (ROS) like ozone (O_3_), hydrogen peroxide (H_2_O_2_), singlet oxygen (^1^O_2_), peroxyl (ROO•) and hydroxyl radicals (•OH), reactive nitrogen species (RNS) like nitric oxide (NO•), peroxynitrite (ONOO-) or peroxynitrous acid (OON). Although each of these species can act on its own, it has been noted a synergistic interaction of the plasma’s constituent.

### 8.1 Mechanisms of action

This technology presents differences in the mechanisms of action according to the food matrix (liquid or solid) or the way of application. When plasma and liquid media are in contact, RONS are transported into the liquid, and secondary active species are created. Therefore, an effect is generated either inside or on the surface of the food as a consequence of the physical and chemical changes triggered by the production of reactive species (Sharma et al., 2018). For solid foods, the application is limited to the surface, and the effectiveness depends on the severity of the treatment, water activity, and porosity. In the last years plasma activated water (PAW), also called plasma acid, plasma activated liquids, nutrients broths (Thirumdas et al., 2018), containing mainly reactive species, has been proposed as an alternative method for fungal reduction.

Reactive species produced by CAP can potentially modify the fungal cell wall and membrane, releasing cytoplasm and leading to cell inactivation. This process can cause the cell to shrink, flatten, and rupture, allowing the cytoplasm and DNA to escape (Los et al., 2018). Furthermore, several oxidizing molecules, such as reactive nitrogen species (RNS) and ROS, accumulate inside the cell and result in cell death. Molina-Hernandez et al. (2022a,b) showed that oxidative stress-dependent reaction cascades triggered by membrane depolarization could account for CAP-induced cell death. In actuality, CAP-generated radicals are poisonous and can act quickly, causing depolarization of the cell and mitochondrial membranes, an increase in intracellular calcium levels (Ca^+2^), DNA damage, and possibly even the induction of cell apoptosis.

**Figure 1:**
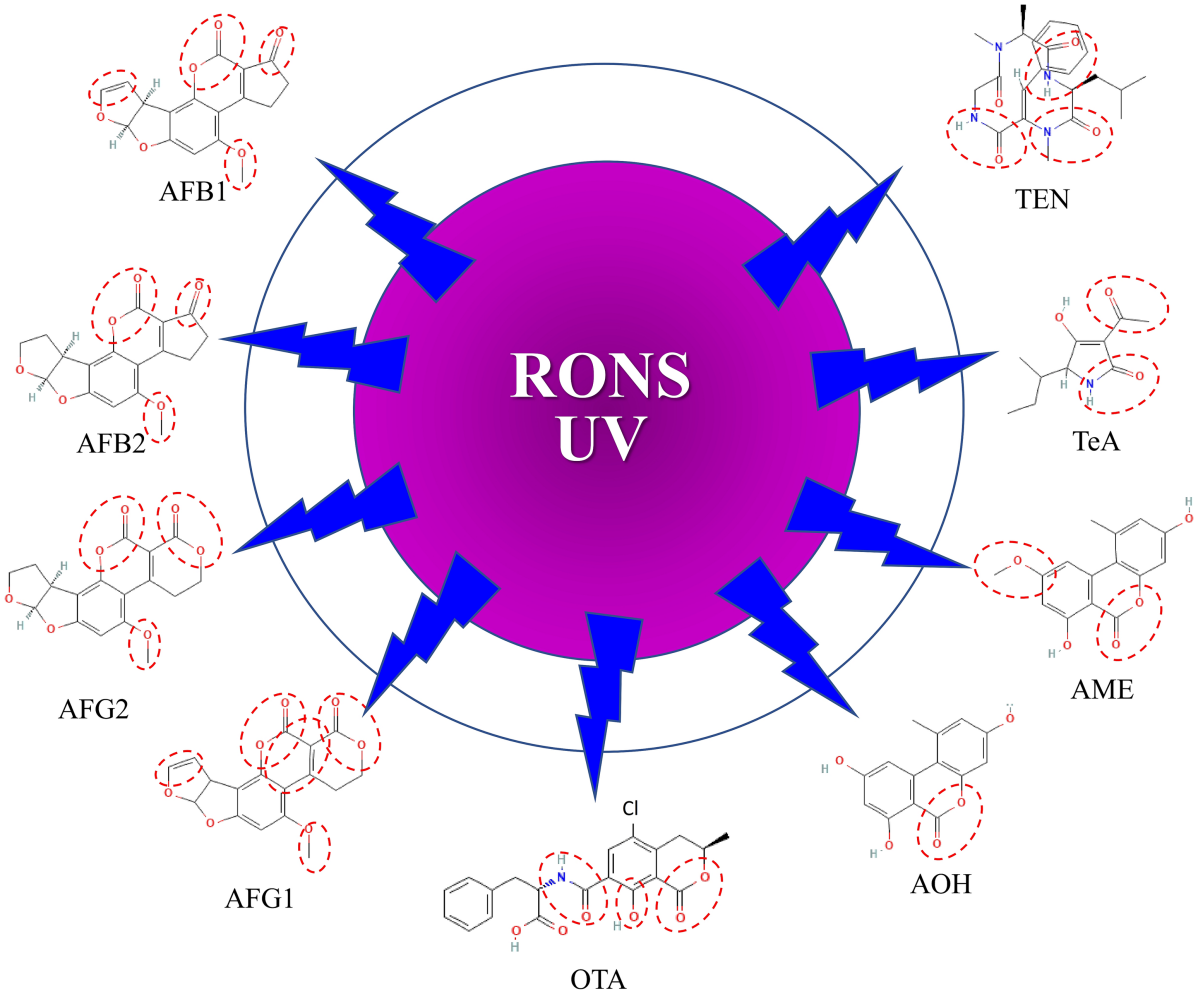
Sites susceptible to attack by reactive oxygen/nitrogen species (RONS) and UV in mycotoxins. AFB1, Aflatoxin B1; AFB2, Aflatoxin B2; AFG1, Aflatoxin G1; AFG2, Aflatoxin G2; OTA, Ochratoxin A; AOH, Alternariol; AME, Alternariol monomethyl ether; TeA, Tenuazonic acid; TEN, Tentoxin.

Mycotoxin degradation is linked to various mechanisms, including chemical interactions with reactive species and UV radiation created by CAP, which results in molecular cleavage by the treatment (Figure 1).

While the mechanisms of AF degradation by CAP are well studied (Wu et al., 2021), those of the other mycotoxins are less elucidated. Neuenfeldt et al. (2023) reviewed potential metabolite products that may be formed after exposure of mycotoxins to CAP treatments. Their results suggest that the by-products of mycotoxin degradation may be non-toxic or have a lower level of toxicity than the parent compound. However, it is not known if foods treated with CAP are safe.

### 8.2 Filamentous fungal suppression and mycotoxin reduction

CAP significantly suppresses the number of fungi and their spores on grain (rice, wheat, corn, barley, and oats) surfaces, and its effect is fungal species dependent. A complete inhibition of *B. cinerea*, *M. fructicola*, and *A. carbonarius* spores and a partial inhibition in *A. alternata* was obtained using an Surface Dielectric Barrier Discharge System (SDBD); with *A. alternata* and *C. carbonarius* the most resistant probably due to the presence of dihydro naphthalene-type melanin (DHN) (Gerin et al., 2018). Also, the diffuse coplanar surface barrier discharge was helpful in reducing *A. niger* and *P. verrucosum* inoculated onto barley (Durek et al., 2018), and to the complete devitalization of *A. flavus, A. alternata*, and *F. culmorum* on maize surface (Zahoranová et al., 2018). Less efficacious resulted in CAP treatment using Dielectric Barrier Discharge (DBD) to control the native microbiota in wheat grains (Los et al., 2018). While PAW was useful for *F. graminearum* inhibition, the primary pathogen for Fusarium head blight (FHB) in spiked samples of wheat (Guo et al., 2022), and it was also useful in field-plot experiments to verify the disease incidence (Ju et al., 2023). On the contrary, no reduction in the grain’s natural fungal diversity and abundance was detected using a corona (Blown-Arc) surface (Kaur, 2023). However, Cold Plasma using O_2_ as input gas was efficacious in reducing fungal frequency and diversity in common and Tartary buckwheat, in particular fungi of genera *Alternaria* and *Epicoccum*, which proved to be the most resistant (Mravlje et al., 2021).

The studies on the efficacy of CAP in groundnuts, nuts, peanuts, and pistachios are concentrated on the reduction of *A. flavus* and *A. parasiticus*, two important species involved in the production of aflatoxins (Esmaeili et al., 2023; Lin et al., 2022), the results showed that the viable spore population is reduced with the increase of treatment duration. CAP also was efficacious successfully in decreasing the percentage disease index of *Colletotrichum musae*, *Fusarium semitectum*, and *Colletotrichum gloeosporioides* responsible of crown rot on Cavendish banana (Sandanuwan et al., 2020), achieving a shelf life of 42 days. Also, the retardation of anthracnose disease caused by *C. gloeosporioides* or *C. asianum* in Nam Dok Mai mangoes was observed after treatment prolonging shelf life by 8 days (Phan et al., 2019). Less effect (25.8% of decay) was observed in blueberry. Sun et al. (2023), using high-throughput sequencing, observed that the fungi surviving to CAP treatment demonstrated stronger biosynthesis and metabolic activity. In Apricot fruits inoculated with *Rhizopus oryzae* and *A. tenuissima*, treatments with DBD at different voltages significantly inhibited the mycelial and reduced spore germination as well as reduced the incidence of soft rot and black spot (81.9 and 82.3%) (Pan et al., 2023). In sun-dried tomatoes, CAP treatments under the NOx regime had a species and dose-dependent effect; in fact, among 32 different species, *A. niger* was the most resistant one. In addition, studies *in vitro* by the same authors revealed that germination kinetics of the fungal spores as a function of CAP time treatment was: *A. chevalieri* < *A. alternata* < *A. tubingensis* < *A. flavus* < *A. niger*. The authors suggested that major hydrophobicity and the presence of melanin contributed significantly to this resistance (Molina-Hernandez et al., 2023).

On the other hand, CAP produced with helium has been used with success to eliminate *A. niger*, *A. westerdijikiae*, *A. steynii*, and *A. versicolor* in coffee beans (Casas-Junco et al., 2019). Also, in naturally contaminated beans (*Phaseolus vulgaris* L.), the CAP treatments effectively inactivated fungal growth (Rüntzel et al., 2019). Very recently, the effects of PAW on *Colletotrichum gloeosporioides*, identified as a serious concern in papaya (Peralta-Ruiz et al., 2023) and chili (Sawangrat et al., 2022), were quite interesting. Compared to previous treatments, the PAW treatment in chili showed a notable 83% increase in efficacy in reducing anthracnose (Sawangrat et al., 2022). In addition, a gliding arc plasma jet (GAPJ) was used to suppress the brown rot disease caused by *A. alternata* in Gala apples; in this case, the results evidenced that 15-min treatment was efficacious in controlling apple brown rot and inhibited the spore germination (Pereira et al., 2024).

Table 3 presents the mycotoxin inactivation results reported in recent years. Our research group recently investigated the potential of CAP in decreasing mycotoxins AFB_1_, AFB_2_, AFG_1_, AFG_2_, and OTA in two different plasma reactive species environments: ozone (O_3_) and nitrogen oxides (NOx). The findings demonstrated that CAP treatments significantly decreased all tested pure mycotoxins: It was found that the degradation of mycotoxins depends on the applied regime, molecule chemical structure, distance from the source, and time-dependent (Laika et al., 2024b). At 60 min and 4 cm from the plasma source, the O_3_ regime was more effective (AFB1 and AFG1 99% decreased, AFB2 and AFG2 60% decreased, and OTA 70% decreased). In naturally contaminated pistachio kernels (*Pistacia vera* L. seeds), the trend was similar. However, the mycotoxins in pistachio were reduced to a much lesser extent than pure molecules. A slightly better response was observed for chopped pistachio kernels than whole ones, most likely due to a larger surface area of the food exposed to plasma (Laika et al., 2024b), implying that the food matrix plays an important role in the CAP degradation process. CAP reduces *Alternaria* toxins such as tenuazonic acid (TeA), one with Alternariol (AOH), Tentoxin (TEN), Alternariol monomethyl ether (AME), and Altenuene (ALT) in dried tomatoes. In particular, the degradation rate under O_3_ regime increased significantly with 60 min of exposure time, reaching 52.80 ± 1.21% of mycotoxin degradation (Laika et al., 2024a). Additionally, GAPJ effectively reduced the production of *A. alternata* mycotoxins, including aAOH, AME and TeA, after 15 min of treatment. The reactive species OH, NO, NO_2_ and NO_3_ are believed to be responsible for these effects (Pereira et al., 2024). Finally, some authors have found that that higher efficacy of CAP was achieved with higher concentrations of mycotoxins (Laika et al., 2024a). The authors hypothesized that many active ions are generated in the plasma during discharge. Therefore, the reaction rate increases with increasing substrate concentration. As a result, the system with the higher initial concentration of mycotoxins showed a faster degradation reaction, eventually leading to greater mycotoxin degradation over time. In addition, Liu et al. (2024) demonstrated that 30-W CAP treatment for 180 s achieved a ZEN degradation of 96.18% in cereals. This degradation was attributed to the oxidative destruction of C=C double bond, resulting in four major degradation products: C_18_H_22_O_7_ (*m*/*z* = 351.19), C_18_H_22_O_8_ (*m*/*z* = 367.14), C_18_H_22_O_6_ (*m*/*z* = 335.14), and C_17_H_20_O_6_ (*m*/*z* = 321.19), that showed a significantly reduced cytotoxicity.

**Table 3:** Summary of the conducted research on cold plasma-induced mycotoxin degradation.

Mycotoxin	Food	Plasma type condition	Principal results	References
AOH, AME, TeA, and Ten	Fresh wolfberries	DBD	Approximately 62.84% of reduction for all mycotoxins	Du et al. (2023a)
DON and OTA	Rice grain	DBD	Reduction of the mycotoxin content by up to 61.25% for DON and 55.64% for OTA.	Guo et al. (2023)
DON	Wheat	DBD	Reduction of the mycotoxin content by up to 25.82%	Chen et al. (2022)
AFT	Peanuts	Rotary plasma jets	Reduction of the mycotoxin content by up to 98.25%	Lin et al. (2022)
DON	Wheat grains	DBD micro discharge	Reduction of the mycotoxin content by up to 56%	Feizollahi et al. (2020)
T-2 and HT-2	Barley grains	DBD	Reduction of the mycotoxin content by up to 43.25% for T2 and 29.23% for HT-2.	Kiš et al. (2020)
OTA	Roasted coffee	DBD with radio-frequency generator (RF)	Reduction of the mycotoxin content by up to 50%	Casas-Junco et al. (2019)
AOH, AME, and TEN	Wheat flour	SDBD	Reduction of the mycotoxin content by up to 60.6% for AOH, 73.8% for AME, and 54.5% TEN.	Hajnal et al. (2019)
AFT	Peanuts	Atmospheric-pressure plasma jet	Reduction of the mycotoxin content by up to 38%	Iqdiam et al. (2020)
AFB_1_	Rice and wheat grains	Corona discharge plasma jet	Reduction of the mycotoxin content by up to 56.6% for rice and 45.7% for wheat.	Puligundla et al. (2020)
AFB_1_ and AFB_2_	Hazelnut blend	Cold atmospheric pressure (AP) and low-pressure (LP)	Reduction of the mycotoxin content by up to 71% for both plasmas	Sen et al. (2019)
AFB_1_ and FB_1_	Maize	DBD	Reduction of the mycotoxin content by up to 65 and 64% for AFB_1_	Wielogorska et al. (2019)
AFB_1_	Corn kernels	High-voltage atmospheric cold plasma	Significant degradation of mycotoxin was achieved in modified atmosphere gas composition and higher RH (40 and 80% instead of 5%).	Shi et al. (2017)

AFB: Aflatoxin B_1_ and B_2_; AOH: Alternariol; AME: Alternariol methyl ether; TeA: Tenuazonic Acid; DON: Deoxynivalenol; OTA: Ochratoxin A; AFT: Aflatoxin; FB_1_, Fumonisin B_1_; AFT: Aflatoxin; TEN: Tentoxin; T-2: Trichothecene; HT-2: Trichothecene.

### 8.3 Drawbacks

Large-scale implementation involves considerable challenges. During the CAP reaction process, numerous distinct types of active substances are created, which can modify the qualitative features of foods. Several reactions, including accelerated lipid oxidation, may affect the sensory properties of CAP-treated foods. Furthermore, it is possible to identify enzyme breakdown, vitamin, and sensory feature loss. The heterogeneity of foods, especially those with high solids or particulate concentrations, can make uniform plasma distribution difficult. The complexity of scaling plasma generation systems to industrial levels and the costs associated with acquiring and operating CAP equipment, including high-voltage power supplies, plasma generation systems, and control systems, represent a significant barrier to its adoption in the food industry. Reproducibility studies of the results are required. Despite these challenges, ongoing research in CAP seeks to optimize this technology.

## 9 Electron-beam (E-beam)

During this process, electrons are generated from electricity in a vacuum. The electrons are fired or pulsed from an electron gun (consisting of a cathode, grid, and anode), creating a beam of electrons. The beam of pulsed electrons is carried across a radio frequency wavelength in the linear accelerator, which has positively and negatively charged cavities that increase the speed of the beam. Unfortunately, its penetration power is low, limiting it to surface disinfection (Hertwig et al., 2018).

### 9.1 Mechanisms of action

Electrons result in breaking molecular bonds and creating free radicals; e-beam may ionize the water molecules to produce unstable free radicals, which can influence either directly the RNA or DNA molecules or indirectly affect the structure of molecules as a result of radiolysis of water, thus damaging other cellular metabolic pathways promoting intracellular oxidation, which results in cell injury and death. According to a recent study (Mohamed et al., 2022), *R. oryzae* cell wall structure was destroyed by E-beam, which also increased chitinase activity and decreased chitin content. The integrity of the cell membrane is compromised, leading to a decrease in pH, an increase in relative conductivity, and a reduction in soluble protein. E-beam induces oxidative stress in cells, raising the concentration of increasing H_2_O_2_ content. This includes the production of free radicals and ROS, which damage membranes and essential enzymes. It also reduces the activity of defense enzymes (CAT and SOD) and DPPH free radical scavenging. This phenomenon suggests that E-beam can induce redox homeostasis disorders, metabolic dysfunction, and cell structural damage.

Microorganisms with large genomes are usually more susceptible to radiation than smaller genomes. Several variables, including dosage, microorganism species, and food composition, frequently influence the e-beam’s ability to decontaminate food. As energetic ions influence the cell membrane and speed up the rate at which microorganisms are destroyed, increasing the irradiation dose can also affect the structure and configuration of RNA (Schmidt et al., 2019).

### 9.2 Filamentous fungal reduction and mycotoxins degradation

The effect of e-beam treatment doses on the inactivation of molds in different food commodities (cereals, beans, and species) has been investigated. Treatments at 6 kGy applied to red pepper powder led to significant decontamination of yeasts and molds naturally present (Woldemariam et al., 2020). Other studies showed a significant decrease of up to 88% in fungal contamination with increasing irradiation doses of up to 6.2 kg. The mixture of fungal subpopulations on maize seeds with varying radiation sensitivity is responsible for the sigmoidal pattern observed in the survival curve of total fungi as determined by the blotter test. According to the authors, the order of the most common fungi’s sensitivity to electron beam treatment was *Penicillium* spp. > *Fusarium* spp. > *Aspergillus* spp., with total inactivation occurring at irradiation doses of 1.7, 2.5, and 4.8 kGy, respectively (Sanaei Nasab et al., 2023). Conversely, in split beans of *Canavalia maritima*, higher doses (10–15 kGy) significantly reduced the fungal incidence and eliminated the mycotoxins. Additionally, beans exposed to 10 kGy radiation had a minimum six-month shelf life (Akhila et al., 2021). However, e-beam irradiation of barley infected with Fusarium spp. at 6–10, kGy did not result in a discernible decrease in the fungal incidence or DON contents. However, it was noted that the resulting barley malt had a much lower DON content and fungal occurrence (Schmidt et al., 2018).

The efficacy of e-beam on the mycotoxins reduction depends on the type of mycotoxin: Yang et al. (2020) reported the effect of ozone and e-beam irradiation on the degradation of zearalenone and ochratoxin A, using a dose of 16 kGy, and found degradation of 92% of OTA and 72% the of ZEN when treated in acetonitrile. Similarly, Luo et al. (2017) observed differences in ZEN and OTA reduction in corn; in this case, the reduction was 56% for ZEN and 75% for OTA. Also, in red pepper, OTA was reduced by 25% after treatments of 30 kGy (Woldemariam et al., 2020). After 20 kGy of E-beam treatment, aflatoxin that was naturally present in maize was significantly reduced, but fumonisin was not (Mohamed et al., 2022).

### 9.3 Drawbacks

The high investment and operating costs associated with electron beam technology represent a significant challenge to its widespread adoption in the food industry. The acquisition and maintenance of electron accelerators, vacuum systems, and radiation shielding entail large investments. In addition, the limited penetration of the electron beam into dense foods requires higher irradiation doses, which can induce undesirable changes in the sensory and nutritional quality of the products. These changes can manifest in color alterations, loss of volatile compounds, protein degradation, and formation of free radicals. Furthermore, the effectiveness of E-beam decontamination is affected by factors such as beam energy, sample geometry, and packaging material properties. Low beam penetrability, especially for low-energy electrons, and the need to irradiate both sides of the product limit its application in large-sized foods or packaging with low beam permeability.

## 10 Ultrasound (US) treatments

The US consists of sound waves whose frequency exceeds the limit of human hearing (around ~20 kHz) and is classified into three groups: (i) power frequency (frequencies between 20 and 100 Hz), (ii) high-frequency US (frequencies between 20 kHz and 100 MHz), and (iii) diagnostic US (frequencies >1 MHz). On the other hand, the US, according to its application, is classified into low intensity (less than 1 W/cm^2^) and high intensity (10–1,000 W/cm^2^) (Gavahian et al., 2021).

### 10.1 Mechanisms of action

**Figure 2:**
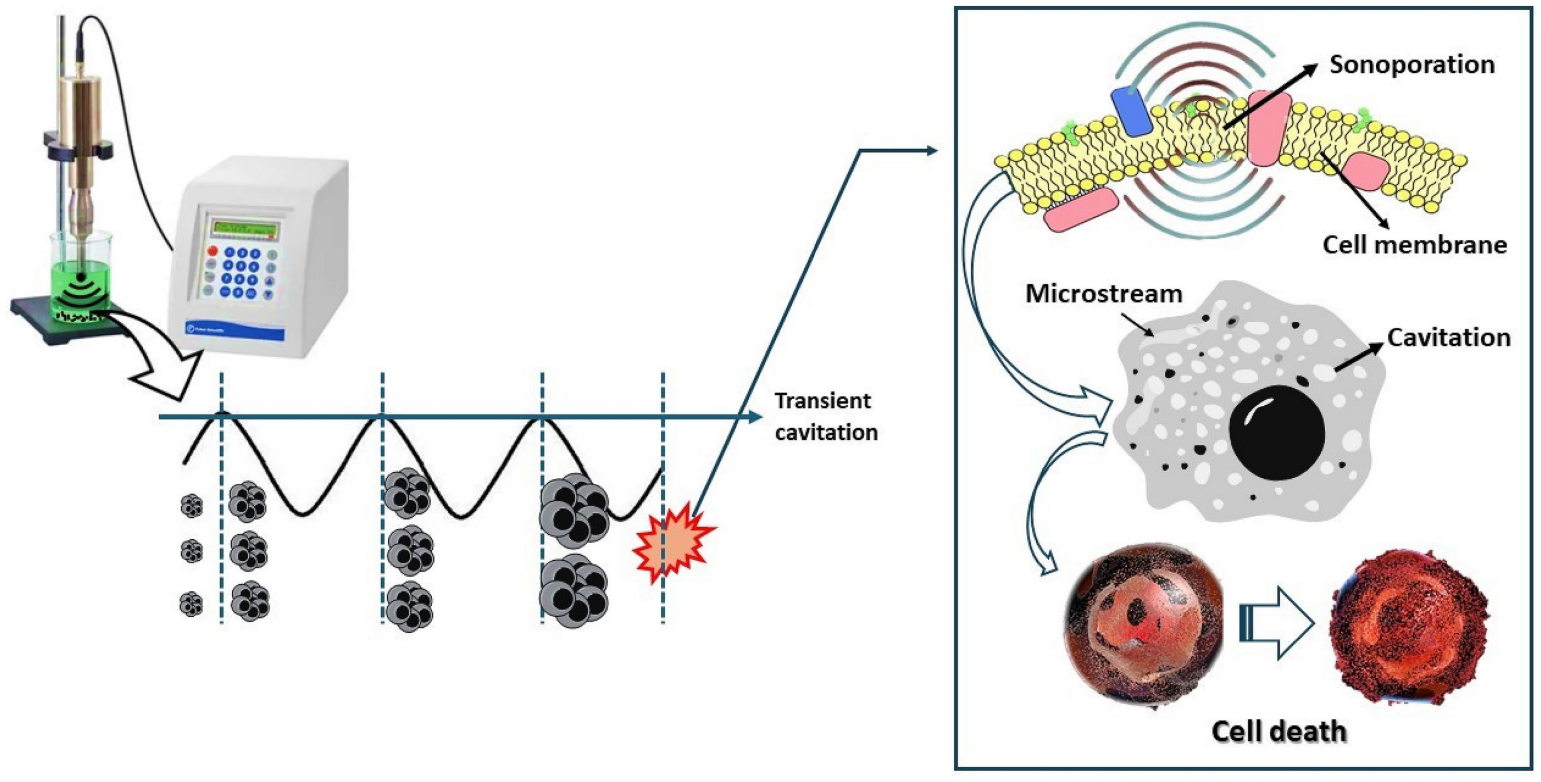
Mechanism of action of ultrasound. Cell death can occur by sonoporation of the cell membrane, by cavitation bubbles, or by microstream.

With high-intensity ultrasound, cavitation bubbles are created through generated pressure cycles, which grow irregularly during the compression/rarefaction cycles, absorbing energy until a maximum when implosively collapsing, releasing a large amount of energy and, in some instances, producing radicals and ROS. This released energy generates shear forces with very high pressure and temperature (5,000 K and 5,000 atm), capable of destroying any membrane or cell wall of microorganisms (Moosavi et al., 2021) (Figure 2). Three aspects influence the effectiveness of microbial inactivation: the cavitation threshold (intensity, frequency, amplitude, temperature, and external pressure), the medium (viscosity, volume, pH, and initial amount of microorganism), and therefore the properties of the microorganism (cell wall, size, shape, endospore or growth phase, and growth phases) (Evelin and Silva, 2020).

There is evidence that using liquids improves microbial inactivation efficacy: higher water content facilitates the transfer of acoustic waves and high energy, increasing the fungicidal capacity (Liu et al., 2019b). In this sense, foods that contain more water, such as fruits and vegetables, are affected by ultrasound treatment to a greater extent. Moreover, it has been mentioned that water breaks down into hydrogen, hydroxyl, and hydrogen peroxide radicals that can break covalent bonds in mycotoxins (Liu et al., 2019b).

### 10.2 Filamentous fungal suppression and mycotoxins reduction

The effectiveness of the US in inhibiting fungi and mycotoxin production has also been reported. Rudik et al. (2020) used high-frequency (24–36 kHz) and low-intensity (~1.0 W/cm^2^) US to reduce fungi and mycotoxins in cereal grains. Villalobos et al. (2019) reported that pretreatment of fresh figs for 30 min at 40 kHz and 60 W combined with osmotic dehydration considerably decreased microbial counts, especially the growth of mycotoxin-producing fungi such as those of the genus *Penicillium* (which produce penicillic acid, griseofulvin, and roquefortine), without affecting the organoleptic parameters of the figs. Nevertheless, Schmidt et al. (2019) reported different findings. They demonstrated that US treatment (45 kHz and 120 W for 5–20 min) on cereal grains had no desired results because it could not prevent fungi’ growth during storage.

In the study reported by Liu et al. (2019b), degradation of AFB_1_ (96.5%), ZEA (95.9%), OTA (91.6%), and DON (60.8%) was achieved using US treatment with intensity between 2.2–11 W/cm^2^ and between 10–50 min. In another study, Liu et al. (2019a) reported a US treatment of 550 W power with a 13 mm-probe, a frequency of 20 kHz, and a power intensity of 6.6 W/cm^2^ for 80 min increased AFB1 degradation by 85.1%. All these studies demonstrate that the US is an adequate treatment to inactivate pathogenic microorganisms in foods without altering the sensory characteristics and stability.

### 10.3 Drawbacks

The attenuation of ultrasonic waves in heterogeneous media, such as food, significantly reduces their penetration, requiring longer processing times to ensure adequate decontamination. This, in turn, can negatively affect the sensory and textural quality of products. Furthermore, the heat generation inherent to the ultrasonic process can degrade thermolabile compounds, such as vitamins and enzymes, compromising the nutritional value of foods. The investment and operating costs of ultrasonic equipment, including generators, transducers, and control systems, are high and can represent an economic barrier to its large-scale implementation.

## 11 Nanoparticles-metal and metal oxide nanostructures-based antimicrobials

Nanomaterials include nanoparticles (NPs), clusters of very thin atoms with dimensions between 1 and 100 nm, and can be classified into organic, inorganic, and carbon-based (Khan et al., 2019). Metallic nanoparticles of Ag, Zn, Pd, Cu, Fe, Se, and Ni were useful as antifungal agents (Cruz-Luna et al., 2021). The size, shape, and structure of metallic NPs are crucial for antifungal activity. It has been observed that the antifungal effect increases with the decrease in size (3–30 nm) since the surface area/volume ratio increases, which allows the fungal cell membrane to be penetrated more easily and affects the cytosol (Win et al., 2020). Larger sizes of nanoparticles (40–90 nm) do not allow cell membrane penetration but affect hyphae and spores, generating malformations in these structures and inhibiting fungal growth (Bocate et al., 2019).

Also, the antifungal efficacy of nanoparticles of metal oxides, such as TiO_2_, CuO, and ZnO, has been reported due to their optical and electrical characteristics (Ajmal et al., 2019). When irradiated with a specific wavelength laser, these NPs can undergo photocatalytic processes and generate antimicrobial effects. In addition, these NPs are considered biocompatible, slightly toxic, very stable, and with greater specificity for their action (Ilyas et al., 2021).

### 11.1 Mechanisms of action

Among metallic NPs, silver NPs (AgNPs) have been the most widely used thanks to their ability to disrupt the cell membrane and other biological functions (Akpinar et al., 2021; Cruz-Luna et al., 2021). In fact, they can alter the structure of proteins, nucleic acids, ribosomes, and gene expression, affecting such essential processes as ATP synthesis, protein synthesis, and nutrient transport (Na^+^ and K^+^), membrane potential and leading to apoptosis (Sunday et al., 2021). Additionally, AgNPs can generate lipid peroxidation due to ROS generated and affect the post-translational modifications of proteins in the Golgi apparatus (Guo et al., 2022; Rizwana et al., 2021). However, antimicrobial activity is also strongly influenced by the size, morphology, surface charge, and type of material with which the NPs are produced. Positively charged NPs react more readily to the negatively charged cell membranes of bacteria and fungi, especially if they are tiny and have a large surface area (Cruz-Luna et al., 2021). Molina-Hernandez et al. (2022a,b) confirmed the adaptive fungal responses during AgNPs with catechin (accumulation of trehalose, glycerol, citric and oxalic acids, volatile compounds derived from the *α*- or *β*-oxidation of fatty acids as alcohols, aldehydes, alkanes, and acids) and several enzymes correlated with cell growth.

**Figure 3:**
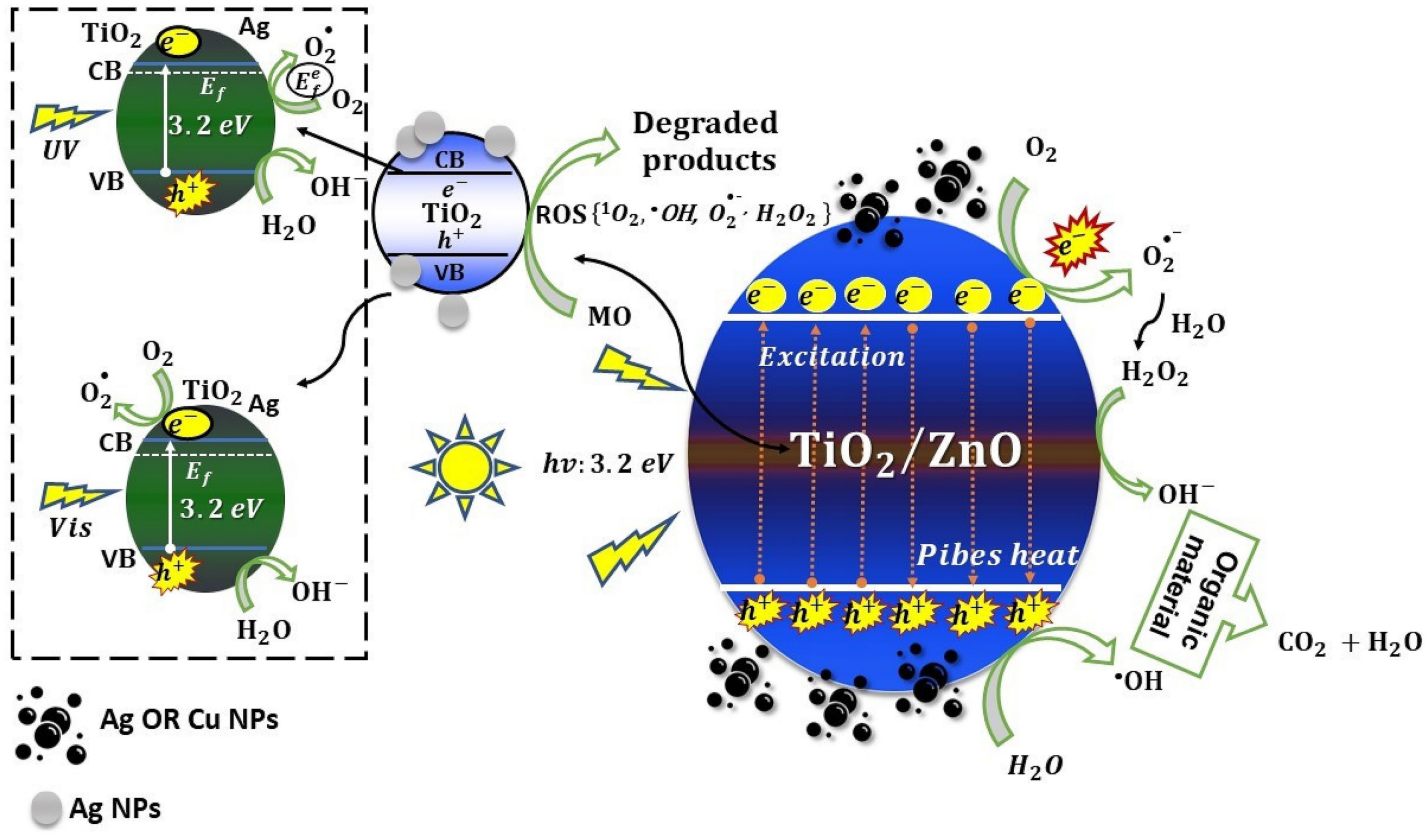
Mechanism of photocatalytic activity of Cu/Ag-doped TiO_2_ or ZnO NPs and ROS production under irradiation.

The antimicrobial activity of metal oxide NPs (TiO_2_, ZnO, CuO) comes from the generation of ROS (Zudyte and Luksiene, 2021), which is a function of the valence band and conduction band energies. Thus, for example, in ZnO, any incident radiation with photons of energy above the bandgap (3.3 eV) will cause the formation of photoactivated free electrons in the conduction band from the valence band and holes in the valence band (Figure 3). The interaction of photogenerated free electrons with environmental oxygen forms superoxide radicals, while the holes generated in the valence band interact with water molecules, producing H^+^ and *OH. Subsequently, the interaction of these radicals with other H^+^ in the medium produces HO_2_* and hydrogen peroxide. Singlet oxygen, a powerful oxidizing agent, can also be produced through aqueous reactions of O_2_^−^ (Ilyas et al., 2021). These radicals are highly reactive and can interact with biomolecules, such as lipids, carbohydrates, DNA proteins, amino acids, and enzymes, on the cell surface, disrupting their vital functions in the cell and causing cell death (Tralamazza et al., 2016).

### 11.2 Filamentous fungal reduction

In agriculture, nanomaterials have offered alternatives for the control of phytopathogenic fungi, especially metallic nanoparticles (Alghuthaymi et al., 2021; Kutawa et al., 2021). In addition, numerous nanomaterials have been created to prevent diseases in citrus, grapes, bananas, apples, mangos, peaches, and nectarine and have demonstrated potential in post-harvest management (Ruffo Roberto et al., 2019).

The antifungal activity of CuNPs with sizes between 11 and 55 nm was studied in tomato crops (*Lycopersicon esculentum*) by comparing their effectiveness with commercial copper-based products (Ashraf et al., 2021). The results showed that a lower concentration was needed in the formulation of antifungals with CuNPs compared to commercial products to achieve a good inhibition of the fungus *Phytophthora infestans* without negatively affecting the plant. Since then, many studies have shown the effectiveness of these nanoparticles against various phytopathogenic fungi (Table 4). On the other hand, nickel nanoparticles (NiNPs) (Ahmed et al., 2016) demonstrated effectiveness against the growth and sporulation capacity of *F. oxysporum* under *in vitro* conditions with 100 ppm of NPs, while under *in vivo* conditions, they reduced the severity up to ca. 60% in tomato and lettuce crops using 50 ppm of NPs. Silver nanoparticles (AgNPs) synthesized with phenolic compounds (PCs) showed antifungal activity against *A. niger* isolated from coffee seeds (Scroccarello et al., 2021), especially on spore germination and mycelial growth, which is in line with the trend of antioxidant capacity (catechin < caffeic acid < myricetin < gallic acid). The strong inhibition was attributed to the ability of the different PCs to generate/stabilize AgNPs with different envelopes, the residual antioxidant capacity, and the ability to interact and aggregate during their “attack” on the hyphae.

**Table 4:** Sample of studies of metal nanoparticles applied for fungi control.

Fungal specie	NP	Synthesis method	Size (nm)	Concentration (μg/g)	Shape	Assay	Antifungal mechanism and MIC	References
*A. niger; A terreus; A. fumigatus*	Cu	Biological (*Talaromyces pinophilus*)	10	2000	Spherical	*In vitro*	ROS. *A. niger* (62. 5 μg/mL); *A terreus* (125 μg/mL); *A. fumigatus* (62.5 μg/mL)	Hasanin et al. (2022)
*A. niger*; *A. oryzae*	Cu	Chemical (hydrothermal)	14 ± 2	0.2–0.8	Spherical	*In vitro*	N.R. *A. niger* (20 μg/mL); *A. oryzae* (20 μg/mL)	Seku et al. (2018)
*F. graminearum; F. poae; F. avenaceum; F. sporotrichioides*	Ag	Biological (*P. ginseng* extracts)	50–90	5–100	Spherical	*In vitro* – *In vivo*	Only a reduction in the sporulation reported	Yugay et al. (2021)
*A. flavus; A. niger; A. tereus; P. notatum; F. solani; F. oxysporum; T. viride; Verticillium dahliae; P. spinulosum*	Ag	Biological (*Bacillus pseudomycoides*)	25–43	10–90	Spherical	*In vitro*	Hyphae damage. *A. flavus* (85 μg/mL); *A. niger* (80 μg/mL); *A. terreus* (80 μg/mL); *P. notatum* (70 μg/mL); *F. solani* (90 μg/mL); *F. oxysporum* (75 μg/mL); *T. viride* (95 μg/mL); *V. dahliae* (75 μg/mL); *P. spinulosum* (75 μg/mL)	El-Saadony et al. (2019)
*Sclerospora graminícola; A. solani; M. phaseolina; Diaporthe longicolla*	Se	Chemical	< 100	0–1,000	Spherical	*In vitro*	ROS. Inactivation of Sulfur proteins in membrane and leakage. Between (150 and 350 μg/mL).	Ismail et al. (2016); Nandini et al. (2017); Vrandečić et al. (2020)
*F. oxysporum* f. sp. *Lactucae; F. oxysporum* f. sp. *Lycopersici*	Ni	RF-thermal plasma system	< 100	50–100	N.R.	*In vitro* – *In vivo*	Inhibition of sporulation and conidia germination. Membrane leakage – DNA, ROS, protein damage. Reduction of 60% of growth with 100 μg/mL.	Ahmed et al. (2016)
*A. flavus*; *F. verticilliodes*	*Zn/CdO: Zn	Chemical (heat treatment)	11.5–29.1	**N.R.	Spherical-Nanorods	*In vitro*	ROS and liberation of Zn^2+^ and Cd^2+^. Cell membrane and organelles leakage. Cell wall penetration and metabolism disturb. *A. flavus* (23% of growth inhibition); *F. verticilliodes* (15% of growth inhibition).	Kumar et al. (2020)
*A. flavus; A. niger; A. fumigatus; A. oryzae; A. Terreus; C. tropicalis;*	HgS	Chemical–physical (hydrothermal-ultrasound)	72.78	100 (ppb)	Crystalline hexagonal	*In vitro*	ROS disrupting cell functions, enzymatic system membrane, growth, morphology, and ATP alterations. N.R.	Ibrahim et al. (2021)

Adapted from Cruz-Luna et al. (2021).

ROS: Reactive oxygen species; N.R: Not reported; R.F: Radio Frequency. ^*^CdO:Zn: Zn nanoparticles doped with CdO. ^**^ N.R.: The final concentration of the NP is non-reported. However, the mol ratio of Zn was reported between 3 and 10 mol% to CdO.

Many studies reported the photocatalytic effectiveness of TiO_2_ and ZnO NPs in inhibiting fungi (Table 5). Using visible radiation (irradiation of visible fluorescence light of 8 W, 2950 lux intensity), Ag-doped TiO_2_ nanoparticles allowed the control of *F. solani* and *Venturia inaequalis* by generating ROS through a photocatalytic mechanism (Boxi et al., 2016). Commercial NPs with sizes less than 100 nm with the capacity to inhibit fungi such as *B. cinerea, P. expansum*, and *F. oxysporum* have been studied, and the control of *B. cinerea* directly in strawberries through a photocatalytic mechanism, using visible light and 10 min of ultrasound to disperse the ZnO NPs has been reported.

**Table 5:** Examples of studies on metal oxide nanoparticles used to reduce filamentous fungi.

Fungal specie	NP	Synthesis method	Size (nm)	Concentration (mg/L)	Shape	Assay	Antifungal mechanism and MIC	References
*Arthrographis cuboidea; A. niger; A. fumigatus*	TiO_2_	Biological (*Mentha arvensis*)	20–70	10,000–30,000	Spherical	*In vitro*	Cell wall penetration. ROS production. DNA, RNA, enzymes, and macromolecules damage. *Arthrographis cuboidea* (10 mg/mL); *A. niger* (10 mg/mL 74% reduction); *A. fumigatus* (10 mg/mL)	Ahmad et al. (2020)
*A. alternata; F. verticilliodes*	ZnO	Chemical (one-pot precipitation synthesis)	65.3	2–5,000	Irregular-shaped	*In vitro*	Zn^2+^ interaction with the fungal cell wall, cell metabolism disturbances, DNA, ribosome disassembly protein denaturation, electron chain disruptions. ROS causing lipid peroxidation. *A. alternata* (5 mg/mL, 34% of inhibition); *F. verticilliodes* (5 mg/mL, 39% of inhibition)	Akpomie et al. (2021)
*B. cinerea*	ZnO	Biological (*Citrus sinensis*)	33.1 ± 11.7	100,000–500,000 (*In-vitro*) – 9.54 Fresh weight of strawberries (*In vivo*)	Hexagonal	*In vitro* – *In vivo*	Size and concentration-dependent effect. Probably cell membrane leakage, cytosolic content affectation, DNA, and protein denaturation. *B. cinerea* (100 mg/mL, 25.2% of inhibition)	Gao et al. (2020)
*P. italicum*	CH/ZnO/SEO	Commercial (Sigma Aldrich)	< 50	Film blends (CH 80/ZnO 10/SEO 10)	Slender shape	*In vitro* – *In vivo*	Loss of membrane integrity of spores. Intracellular interaction, ionic membrane imbalance, DNA denaturation. Cell membrane destruction, ROS. Fungal cell wall synthesis inactivation. Inhibition of 85% growth and almost 80% spore germination inhibition.	Wardana et al. (2022)
*F. oxysporum* f. sp. *lactucae*	CuO-embedded hydrogels	Commercial (Sigma Aldrich)	< 50	31	Spherical	*In vitro* – *In vivo*	ROS. Membrane disruption, cell lysis. Up to 74.1% reduction of dehydrogenase activity.	Shang et al. (2021)
*P. italicum; P. digitatum*	CH/CuO/CEO Films	Commercial (Sigma Aldrich)	< 50	6–75 NPs; Film blends (CH 80/CuO 10/CEO 10)	Slender shape	*In vitro*–*In vivo*	Spore growth is inhibited. Loss of membrane integrity. Metabolic disruption. Synergistic effect between CuO and CEO. Membrane disruption and more effective penetration of CuO. Up to 70% inhibition of spores and up to 80% inhibition of mold infection in vivo.	Wardana et al. (2021)
*Penicillium* spp.; *A. flavus*	GO/CuO	Chemical (hydrothermal and Hummer’s method)	<100 lengths and 23 size	500–1,000	Cuo (nanorods)/ GO (nanosheets)	*In vitro*	ROS. Membrane penetration of ion release. *Penicillium* spp. (500 μg/mL); *A. flavus* (1,000 μg/mL)	Banu et al. (2022)
*A. niger*	MgO/Cellulose	Chemical (co-precipitation)	21	2,000–8,000	Spherical	*In vitro*	The nanocomposite between cellulose and MgO NPs prevents the agglomeration of NPs, improving the active surface available. Damage to fungi’s membranes. Induction of oxidative stress. Release of toxic ions of Mg2+, membrane leakage. Affectation of cytosolic content. The composite formed by 4 mg/mL MgO and 1 mg/mL cellulose inhibited growth by 85.03%.	Safaei and Taran (2022)
*A. fumigatus; A. niger; A. cuboidea*	CaO	Biological (pomegranate peel extract)	30–50	10,000–30,000	Spherical	*In vitro*	ROS causes protein, enzymes, and DNA denaturation, affecting several cell processes and causing death. *A. fumigatus* (30 mg/mL, 19.6% of inhibition); *A. niger* (30 mg/mL, 18.5% of inhibition); *A. cuboidea* (30 mg/mL, 29.6% of inhibition)	Ahmad et al. (2022)

N.R: Not reported; US: Ultrasound; CH: Chitosan; SEO: Indonesian sandalwood essential oil; CEO: Indonesian cedarwood essential oil; GO: Graphene oxide.

Despite a large number of published reports on the use of metal NPs and metal oxides (Table 5), few studies of *in situ* NPs demonstrate these systems’ applicability directly in foods (Gao et al., 2020; Luksiene et al., 2020; Maringgal et al., 2020; Rezaei et al., 2020; Shang et al., 2021; Wardana et al., 2021; Zudyte and Luksiene, 2021).

### 11.3 Drawbacks

Metal and metal oxide nanoparticles safety, especially at high concentrations, is a general concern; since metal and metal oxide nanoparticles can alter the structure of proteins, nucleic acids, ribosomes, and gene expression. It is essential to assess the cytotoxicity in different cell lines or microorganisms and decide the safety of the nanoparticles used in different environments. Very low doses of metal oxide nanoparticles are preferred to avoid cytotoxicity in non-target microorganisms or cells. Nanoparticles’ complete and accurate characterization is complex due to their small size and the diversity of available analytical techniques. In the food matrix, nanoparticles tend to agglomerate due to intermolecular forces, which hinder their dispersion and affect their properties.

## 12 Concluding remarks

The non-thermal technologies discussed here demonstrated the potential of reducing mycelium proliferation, preventing spore propagation, and degrading mycotoxins during post-harvest storage of cereals, seeds and fruits. The outcomes of the different studies indicate that these non-thermal technologies decrease fungal proliferation by altering the structure of the wall and cell membrane and the DNA structure, disrupting fungal metabolism and leading to rapid microbial cell death. In addition, it is evident from the comparative analysis of the antifungal activity of the non-thermal technologies reported here that they share several similarities, notably in the production of ROS, which induces oxidative stress and cell death, binds DNA, proteins and enzymes to disrupt cell function, prolongs the lag phase associated with microbial proliferation, and reduces the growth rate. At the same time, these technologies use a multi-targeted mode and prevent microorganisms from developing resistance; some of these technologies, like CAP, PL, and UV light, are limited to a surface-level treatment. Therefore, it is to consider that the small proportion of alive cells that remain can overcome the stress induced by ROS, producing enzymes and compounds like glutathione as well as osmolytes like trehalose that protect lipid membranes, stabilize proteins and act as free radical scavengers, and modulate cell wall carbohydrates such as chitin and glucan. Consequently, it would be very difficult for the organism to mount a stress response, even if it had enough time. It is also important to remember that stress exposure may strengthen memory or priming, making them more resilient to stress in the future.

As far as the detoxification of mycotoxins is concerned, the non-thermal processing methods discussed here employ diverse mechanisms for detoxifying mycotoxins. Certain as approaches, including CAP, E-Beam, and O_3_, generate reactive species and deactivate the mycotoxins by altering their structure. Other techniques, such as PL and UV, instigate the fragmentation of the mycotoxins via photochemical reaction and destroy their molecular structure, which is responsible for their toxicity. However, several uncertainties remain regarding the toxicity of the degradation products generated by these treatments within the food matrix. While some authors reported that the degradation products are less toxic that the original molecule, further research is needed to fully understand their safety. For instance, CAP treatment of ZEN resulted in less toxic compounds (Liu et al., 2024). Similarly, CAP treatment of OTA in coffee produces slightly toxic compounds, such as L-phenylalanine, and patulin which can be completely decomposed by O_3_ in diglycolic acid, oxalic acid, and CO_2_, (Casas-Junco et al., 2019), Additionally, The main ozonolysis products of AFB1 including C_17_H_14_O_10_, C_18_H_16_O_10_, C_16_H_10_O_6_, C_19_H_15_NO_9_, C_17_H_12_O9, and C_17_H_12_O_9_ did not show any toxic effects on animals (Afsah-Hejri et al., 2020).

Even though these non-thermal technologies have numerous benefits for the food industry, some are still exclusively utilized in laboratories and are seldom employed in the field. The total inactivation of spores with some of these technologies (CAP, O_3_, PL) could require a long, prolonged treatment time, which could compromise the color, the integrity of lipids, proteins, and vitamins, and the texture of the product’s texture. Thus, for these technologies to be used successfully, it is crucial to identify key parameters that influence both food quality and the inactivation of molds and mycotoxins to optimize the process. However, defining a precise dose–response range is challenging as various factors such as the fungal species, the amount of inoculum, the spore or mycelium structure, and whether the fungi are xerophilic or acidophilic all play a role. The equipment used, such as the CAP, and the presence of certain gases can also influence the effectiveness. The composition of the food, water content, water activity, and structure also play an important role, with treatments generally more effective in liquid than solid media. Additionally, the shape of the samples is a challenge for the application as many of these technologies cannot treat samples with irregular forms uniformly, making large-scale processing challenging. Therefore, one of the most important challenges is to optimize the parameters for non-thermal technologies since it is a complex task that requires careful matching of the technology, food characteristics, and specific mycotoxins and fungi. For this reason, the successful application of these methods in post-harvest scenarios depends on achieving a balance between treatment efficacy and food quality preservation. Further research is necessary to understand the long-term effects on the sensorial and nutritional properties of the treated products as well as the potential allergenicity in these products. In the case of nanoparticles, it is important to study their migration, toxicity, and permissible limit when using them in foods or food packaging containing nanoparticles.

## 13 Future trends

The future of non-thermal technologies to eliminate fungal growth and mycotoxin contamination in food is promising. Innovations in this area could include the use of combined non-thermal technologies and natural antifungal compounds to increase the efficacy of fungal control. This approach could lead to the development of safer, more natural methods of food preservation and reduce the need for synthetic chemicals.

Although the ability of microorganisms such as lactic acid bacteria and yeasts to biotransform or adsorb mycotoxins in fermentative processes is well known, synergy with non-thermal low-intensity technologies such as pulsed electric fields, cold plasma, and ultrasound represents an emerging and promising area of research. By enhancing cell permeability, increasing enzymatic activity, and facilitating the transfer of metabolites from beneficial microorganisms, these technologies offer the under-researched potential to reduce mycotoxin contamination in food, thereby preserving nutritional quality and improving food safety.

Studies on the biology of spore resistance, dormancy, and germination could lead to new approaches for food preservation that take advantage of the signaling pathways associated with these phenomena or provide insights into the precise elimination of spore subpopulations. Therefore, it is only logical to further investigate the responses of fungi to sublethal stress caused by these technologies. In addition, global studies have shown that although non-thermal technologies can effectively reduce mold contamination and infections caused by these microorganisms, their effects on mold growth, biosynthesis, and mycotoxin degradation vary depending on the fungal species and mycotoxin structure. Therefore, a large number of fungi still need to be investigated. This area will grow as more and more countries introduce legislation to regulate the handling of mycotoxins and fungal spores. On the other hand, it is important to study microorganisms resistant to harsh environmental conditions, as they are more resilient to various treatments and can provide insights into developing of more robust non-thermal methods. Understanding their resistance mechanisms can lead to innovative solutions that increase product safety and efficacy while minimizing environmental impact. In addition, research into the genetic and metabolic pathways of these microorganisms could reveal new targets for intervention and enable the development of specialized treatments that are both effective and sustainable.

Developments in materials science and engineering may result in discoveries that increase the efficiency of these technologies and open the door for more ecologically friendly solutions.
